# DLK signaling in axotomized neurons triggers complement activation and loss of upstream synapses

**DOI:** 10.1016/j.celrep.2024.113801

**Published:** 2024-02-14

**Authors:** Elham Asghari Adib, Jennifer L. Shadrach, Lauren Reilly-Jankowiak, Manish K. Dwivedi, Abigail E. Rogers, Shameena Shahzad, Ryan Passino, Roman J. Giger, Brian A. Pierchala, Catherine A. Collins

**Affiliations:** 1 Department of Molecular, Cellular and Developmental Biology, University of Michigan, Ann Arbor, MI, USA; 2 Department of Biologic and Materials Sciences, University of Michigan, Ann Arbor, MI, USA; 3 Department of Cell and Developmental Biology, University of Michigan Medical School, Ann Arbor, MI, USA; 4 Department of Anatomy, Cell Biology and Physiology, Indiana University School of Medicine, Indianapolis, IN, USA; 5 Department of Neurosciences, Case Western Reserve University, Cleveland, OH, USA; 6 These authors contributed equally; 7 Lead contact

## Abstract

Axotomized spinal motoneurons (MNs) lose presynaptic inputs following peripheral nerve injury; however, the cellular mechanisms that lead to this form of synapse loss are currently unknown. Here, we delineate a critical role for neuronal kinase dual leucine zipper kinase (DLK)/MAP3K12, which becomes activated in axotomized neurons. Studies with conditional knockout mice indicate that DLK signaling activation in injured MNs triggers the induction of phagocytic microglia and synapse loss. Aspects of the DLK-regulated response include expression of C1q first from the axotomized MN and then later in surrounding microglia, which subsequently phagocytose presynaptic components of upstream synapses. Pharmacological ablation of microglia inhibits the loss of cholinergic C boutons from axotomized MNs. Together, the observations implicate a neuronal mechanism, governed by the DLK, in the induction of inflammation and the removal of synapses.

## In brief

Following peripheral nerve injury, microglia hug the cell bodies of axotomized motoneurons, which lose their upstream synaptic inputs. Asghari Adib et al. demonstrate that the phagocytosis of synaptic components by microglia is governed by the DLK kinase, which becomes activated in injured neurons.

## Graphical abstract

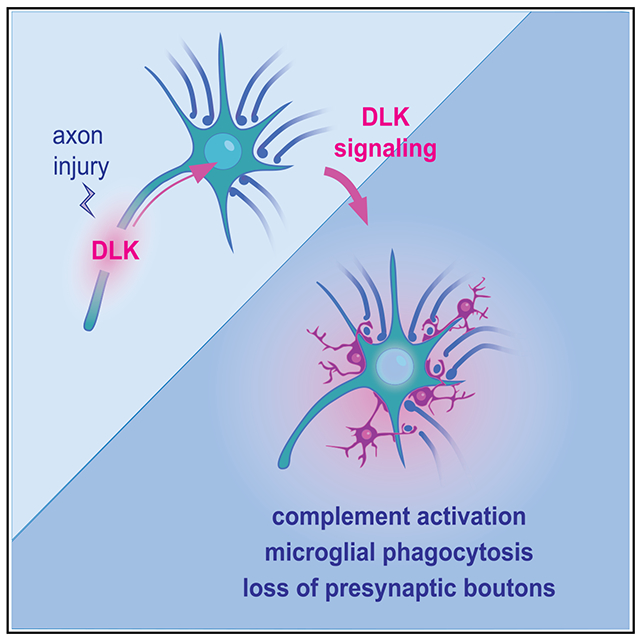

## INTRODUCTION

Physical loss of synapses is an important form of structural plasticity in the nervous system. In many cases during developmental pruning, responses to injury, and neurodegenerative disease, synaptic components are phagocytosed by microglia. The upstream triggers that recruit microglia, and engulf some synapses but not others, are not well understood.

Here, we investigate one of the first-discovered forms of microglia-associated synapse loss: the loss of presynaptic inputs onto axotomized motoneurons (MNs) following peripheral nerve injury (PNI). This type of synapse loss was originally coined “synaptic stripping,” based on the observation that microglia interdigitate their processes between the cell bodies and presynaptic boutons, suggesting an active role in “lifting” or removing synapses from the axotomized MN.^1,2^ However, whether microglia actually play an active role in axotomy-driven synapse loss has been questioned,^1,3^ since previous manipulations aimed at reducing the population of microglia in the spinal cord have not yielded strong impairments to the global loss of presynaptic markers from injured MNs.^4–6^ Rather than an executory role by microglia, it has been proposed that synaptic loss may be initiated by events that occur within the axotomized neuron.^1^ However, there are to date no factors known to be required within neurons to initiate stripping.

We considered the neuronal dual leucine zipper kinase DLK (MAP3K12) to be an attractive candidate to regulate synapse loss from axotomized MNs. Studies in multiple model organisms and paradigms of axonal injury have identified DLK as a sensor of axonal damage.^7–12^ DLK is transported in axons and signals retrogradely^10,13^ to mediate transcriptional responses in the cell body that are associated with varied responses, including axonal regeneration^9,14,15^ and neuronal death.^7,8,16–20^ In addition to cell-autonomous responses to axonal damage, a handful of studies have also noted roles for DLK in neuroinflammation in mouse models of neuropathic pain^21–23^ and amyotrophic lateral sclerosis (ALS).^24^

Using mice conditionally deleted for DLK in MNs, we found that DLK concomitantly orchestrates synapse loss and microglial inflammation from axotomized MNs in the lumbar spinal cord following sciatic nerve crush. RiboTag profiling of axotomized MNs identified that downstream targets include upstream regulators of the classical and lectin pathways of complement. We found that DLK triggers C1q expression first from the axotomized MN, and then later in surrounding microglia, which subsequently phagocytose presynaptic components of upstream synapses. These findings shed light on the mechanism(s) of synapse loss from axotomized neurons and implicate DLK signaling as a regulator of microglial responses from damaged neurons. Because DLK is known to become activated in various paradigms of neuronal stress and injury,^7,8,24–27^ this work brings a new view of DLK as an attractive candidate regulator of complement activation and synapse loss in multiple scenarios of injury and disease.

## RESULTS

### Neuronal DLK is required for axotomy-induced synapse loss

**Figure 1.**
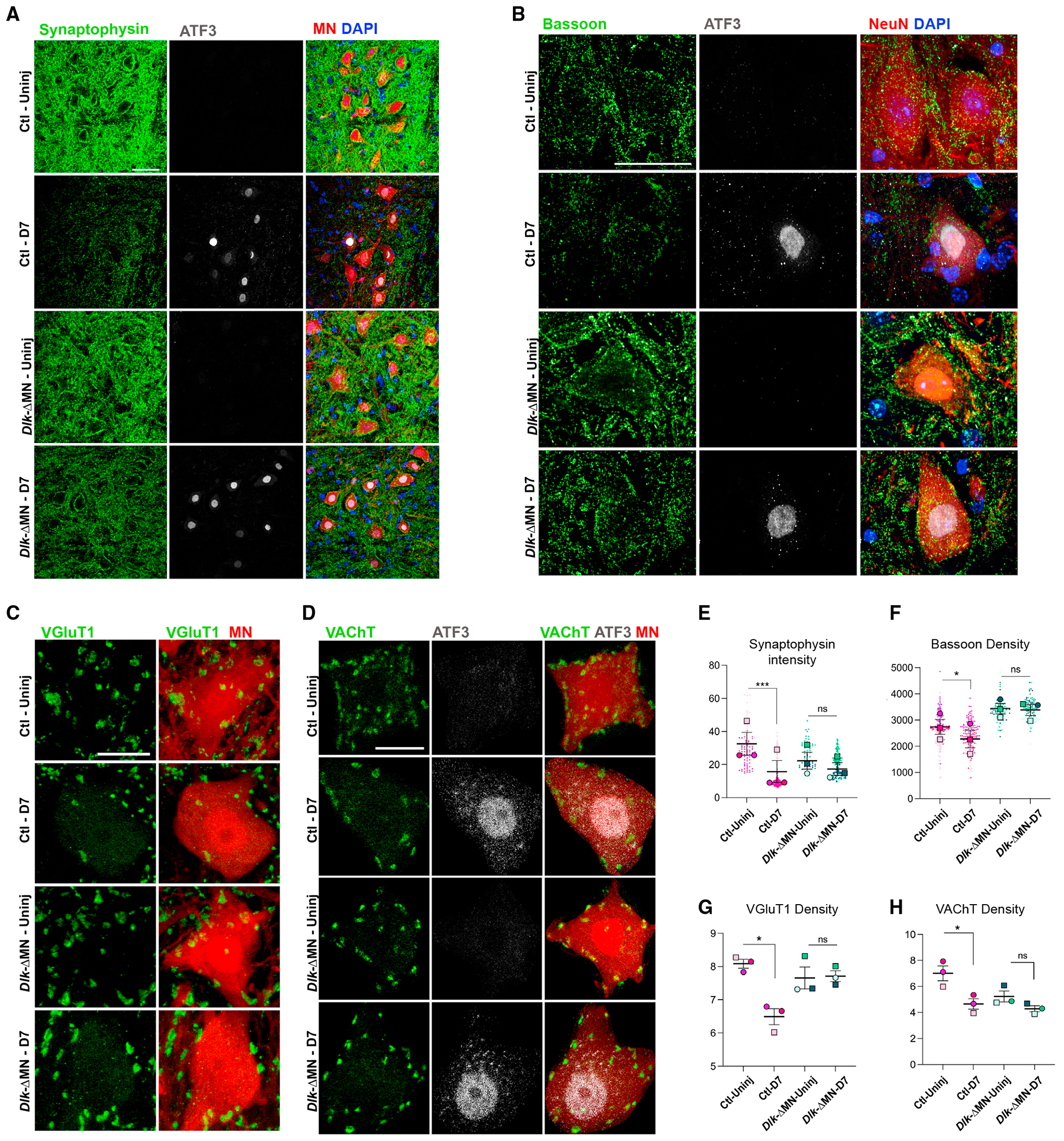
Neuronal DLK influences axotomy-induced synapse loss (A) Synaptophysin (green) surrounding motoneurons (MNs) labeled by Rosa26-tdTomato in *ChAT-Cre; Rpl22*^HA/HA^;Ai14/Ai14 mice, which are either Ctl (*Dlk*^+/+^) or *Dlk-ΔMN* (*Dlk* KO, *Dlk*^fx/fx^). MNs in the L3–L6 lumbar segments of the ventral spinal cord were examined 7 days post sciatic nerve crush (SNC). Axotomized MNs on the side IL to the injury site (D7) are identified by their expression of ATF3 (gray), while uninjured (Uninj) MNs are observed on the CL side of the same longitudinal tissue section. Scale bar, 50 μm. (B) The presynaptic active zone component Bassoon (green), surrounding MNs (labeled by NeuN) in the L3–L6 lumbar segments of the ventral spinal cord 7 days post SNC (D7). Genotypes are Rpl22^HA/HA^; *Dlk*^+/+^; *ChAT-Cre* for Ctls and *Rpl22*^HA/HA^; *Dlk*^fx/fx^; *ChAT-Cre* for *Dlk-ΔMN*. Injured MNs in the injured side of spinal cord sections are identified based on ATF3 staining (gray), Scale bar, 50 μm. (C) Quantification of synaptophysin intensity (shown in A) surrounding the MN cell body surface. The entire pool of MNs in L3–L6 lumbar segments were imaged from four or five longitudinal sections from n = 3 mice per genotype. Small dots indicate the mean synaptophysin intensity measured for each MN (sum green pixel intensity/area μm2 of tdTomato), while large dots indicate the mean of these measurements per mouse. Paired t test was done between Ctl injured and Ctl uninjured samples. ***p = 0.0002. The same test was done for *Dlk-ΔMN* injured and uninjured conditions. (D) Quantification of Bassoon density. The intensity of Bassoon was measured within a 20-μm distance from the surface of the neurons for the entire MN pool in L3–L6 lumbar sections. Small dots indicate the measurements for individual neurons, while large dots indicate the mean of these measurements per mouse. n = 3 per genotype. Paired t test was done between Ctl injured and Ctl uninjured samples. *p = 0.01. The same test was done for *Dlk-ΔMN* injured and uninjured conditions. (E) VGlut1 (green) presynaptic afferents on MN cell bodies (tdTomato) in uninjured and 7 days post SNC. Genotypes are Ai14/Ai14; *Rpl22*^HA/HA^; *Dlk*^+/+^; *ChAT-Cre* for Ctls or Ai14/Ai14; *Rpl22*^*HA/HA*^; *Dlk*^*fx/fx*^; *ChAT-Cre* for *Dlk-ΔMN*. Scale bar, 20 μm. (F) Immunohistochemistry of ventral spinal cord 7 days post SNC (D7) for VAChT (green), a component of C boutons along with ATF3 (gray), and tdTomato (red). Genotypes are Ai14/Ai14; *Rpl22*^*HA/HA*^; *Dlk*^+/+^; *ChAT-Cre* for Ctls and Ai14/Ai14; *Rpl22*^*HA/HA*^; *Dlk^fx/fx^; ChAT-Cre* for *Dlk-ΔMN.* Scale bar, 20 μm. (G) Quantification of mean VGlut1 synaptic density on MN cell bodies. From 20-μm confocal stacks of MN cross sections, the number of VGlut1 boutons counted on each MN cell body was normalized to the cell body perimeter and reported as per 100 μm. The mean synaptic density for visible MNs in segments L3–L6 (totaling 100–150 MNs per animal) is plotted for each animal. A one-way ANOVA with the Tukey test for multiple comparisons was performed. *p < 0.05. (H) Quantification of mean VAChT bouton density on MN cell bodies. The number of VAChT boutons normalized to perimeter was measured for all MNs in L3–L6 within the CL (uninjured) and IL (injured) sides of three sagittal sections per animal. The mean is shown for each animal, n = 3 animals per condition. A one-way ANOVA with the Tukey test for multiple comparisons was performed. *p < 0.05, and ****p < 0.0001.

To ask whether DLK is required cell autonomously in motoneurons (MNs) for axotomy-triggered synapse loss, we used previously characterized *Dlk* conditional knockout (KO) mice,^28^ together with *ChAT-IRES-Cre* to selectively delete DLK in MNs. We refer to these mice as *Dlk-ΔMN* in this text. While global *Dlk* KO mice die soon after birth,^15,29,30^ *Dlk-ΔMN* mice developed normally and did not exhibit overt phenotypes (Figures S1A and S1B). However, these mice had an increased number of motoneurons (Figures S1C and S1D), likely due to a role for DLK in developmental neuronal death.^31^ We assessed the presence of multiple synaptic markers on the cell body of MNs by confocal microscopy. We used the Ai14 Rosa-tdTomato reporter and NeuN staining to delineate the surface of the MN cell body. We imaged injured MNs and their uninjured counterparts from equivalent locations in the ipsilateral (IL, injured) and contralateral (CL, uninjured) sides of the same tissue sections following unilateral sciatic nerve crush (SNC). Activating transcription factor 3 (ATF3), which becomes induced specifically in axotomized MNs,^32^ confirmed the identification of injured MNs (Figures 1A, 1B, and 1D).

In control (Ctl) animals, within 7 days post SNC, the cell body surface and surrounding area of axotomized MNs showed a dramatic loss in synaptophysin (Figures 1A and 1E). This abundant and ubiquitous presynaptic protein is expected to be present in most if not all of the presynaptic boutons that make synapses with MNs, and the loss of synaptophysin staining in the region of axotomized MNs has been described in previous studies.^33–35^ This loss in synaptophysin was strongly reduced in *Dlk-ΔMN* mice (Figures 1A and 1E).

We then examined the presence of presynaptic cytomatrix protein, Bassoon, surrounding injured and uninjured MNs. Bassoon coats the surface of uninjured MNs with a uniform density. Seven days post SNC, Ctl MNs show a reduction in Bassoon density on the cell body surface (Figures 1B and 1F). Compared to Ctl, we observed a higher density of Bassoon surrounding both injured and uninjured MNs in *Dlk-ΔMN* mice. Strikingly, injury caused no significant change in Bassoon density or distribution surrounding MNs deleted for *Dlk* (Figures 1B and 1F).

We also examined the distribution of specific synapses post SNC. Vesicular glutamate transporter 1 (VGlut1) is expressed at afferent synapses made by muscle spindle Ia axons.^36,37^ Ctl MNs showed a 20% reduction in VGlut1 synapse density 7 days post SNC. In contrast, MNs in *Dlk-ΔMN* mice showed no significant reduction (Figures 1C and 1G). The cholinergic C-bouton inputs from V0c interneurons, visualized by staining for vesicular acetylcholine transporter (VAChT) showed a 28% reduction in density on the cell bodies of injured MNs in Ctl mice. In contrast, MNs in *Dlk-ΔMN* mice showed a reduced density of VAChT synapses, which did not significantly change following axotomy (Figures 1D and 1H).

## Neuronal DLK is required for microglial response around axotomized MNs

**Figure 2.**
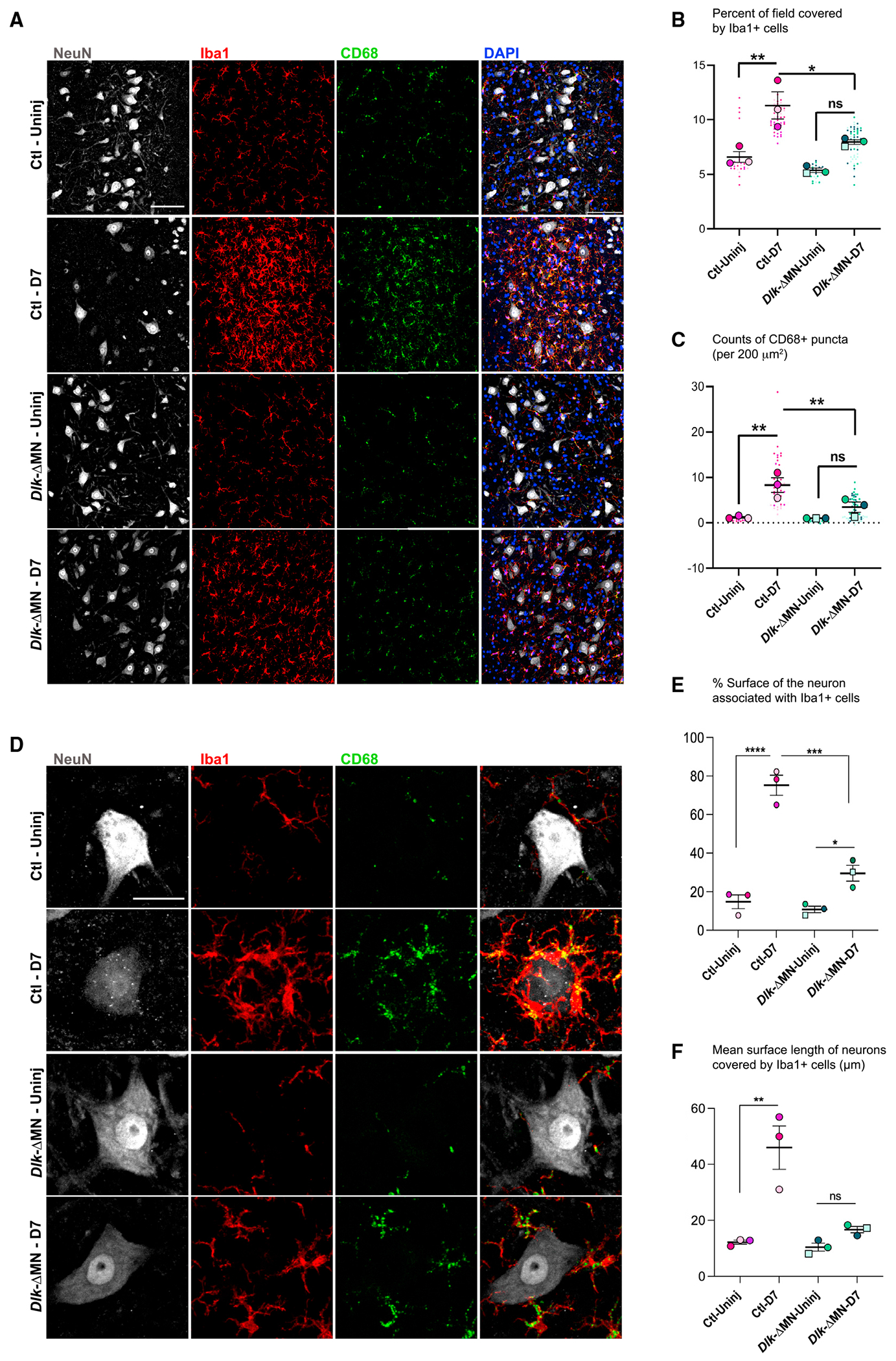
Neuronal DLK influences microglial responses to PNI (A) Iba1 (Red) and CD68 (green) indicate microglia/monocytes and their phagocytic lysosomes increase in density surrounding injured motoneurons (labeled by NeuN in gray) 7 days post SNC. This response is attenuated in *Dlk-ΔMN* animals. Genotypes are *Rpl22*^*HA/HA*^; *Dlk*^+/+^; *ChAT-Cre* for Ctls and *Rpl22*^HA/HA^; *Dlk*^fx/fx^; *ChAT-Cre* for *Dlk-ΔMN.* Scale bar, 200 μm. (B) The percentage of the field of view (within 780 × 780 μm^2^ images of injured/uninjured MNs) that was Iba1+, indicating the presence of microglia. Images were collected from four or five longitudinal slices that covered the entire MN pool in L4–L6. (C) CD68^+^ puncta, indicating the presence of microglial lysosomes, were counted in both injured and uninjured sides of the spinal cords (in the same images described for B) and were graphed based on counts of CD68 puncta per 200 μm^2^. (D) Close-up views of Iba1+ cells interacting with axotomized motoneurons. Scale bar, 20 μm. (E and F) Extent of the motoneuron surface contacted by microglia at D7. From merged images of individual MNs, a line tool was used to measure the fraction of MN perimeter that is in physical contact with microglia. (F) The mean length of individual lines, which indicate the length of MN surface contacted by a single microglial process. Statistical comparisons were evaluated for mean values per animal (n = 3 per genotype) using one-way ANOVA with the Tukey test for multiple comparisons. *p < 0.05, **p < 0.005, ***p < 0.005, and ****p < 0.0001.

Previous studies of synaptic stripping from axotomized MNs have hypothesized and debated the role of microglia in this synaptic loss.^1,38^ We therefore asked whether the microglial response was altered around *Dlk* KO MNs. We probed the IL and CL ventral horn containing injured and uninjured MNs for the microglia and macrophage marker ionized calcium binding adapter molecule 1 (Iba1), and CD68, which is upregulated by phagocytic microglia.^39–41^ In Ctl animals, SNC induces a large increase in Iba1+ cells (Figures 2A and 2B) and CD68 puncta (Figures 2A and 2C) surrounding injured MNs in the IL spinal cord. This increase also occurred in *Dlk-ΔMN* animals; however, the degree was less pronounced (Figures 2A–2C, also shown in Figures 5C and 5D). More strikingly, loss of DLK in MNs affected the close interactions made by microglia with MNs. In Ctl animals, Iba1+ cells tightly hug the cell body of axotomized MNs. In *Dlk-ΔMN* animals, Iba1+ cells still make contact with axotomized MNs but the extent of contact is greatly reduced (Figures 2D–2F). Together, these data suggest that specific microglial responses to axotomized MNs require cellular events regulated by DLK function in MNs.

### Addressing the role of microglia in synaptic loss

To further address the role of microglia in axotomy-triggered synapse loss, we used the Csf1r inhibitor PLX5622 to ablate microglia. Adult (3 months old) Tmem119-EGFP transgenic mice were fed chow formulated with PLX5622 for 2 weeks prior to SNC and the following 7 days before sacrifice. This treatment led to a 20-fold reduction in density of EGFP-positive cells surrounding injured MNs and a 6-fold reduction around uninjured MNs (Figures S2A and S2B). In mice raised on Ctl chow, injury induced a reduction in C-bouton density that reproduced our observations in Figures 1D and 1H. However, in mice treated with PLX5622, we observed no significant change to C-bouton density (Figures S2C and S2D). We interpret that microglia play an important role in the synaptic changes induced by nerve injury.

### RiboTag profiling of DLK-regulated responses in injured MNs

**Figure 3.**
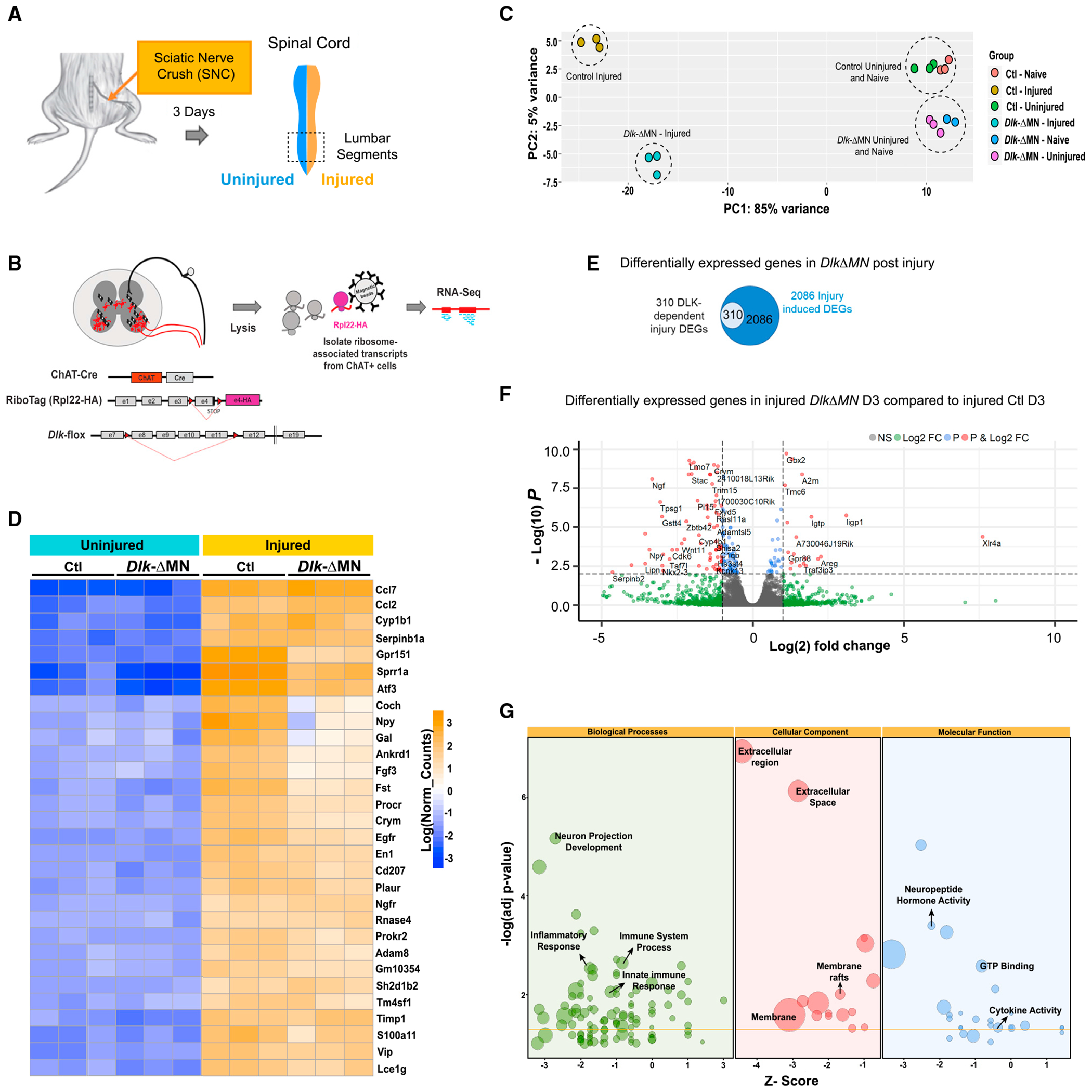
RiboTag profiling of DLK-regulated responses in injured MNs (A) Unilateral SNC was performed at mid-thigh level. The injured and uninjured side of the lumbar spinal cord was collected in the same mice 3 days post SNC. We also collected spinal cords from naive, completely uninjured mice. Genotypes are *Rpl22*^HA/HA^; *Dlk*^+/+^; *ChAT-Cre* for Ctls and *Rpl22*^HA/HA^; *Dlk*^fx/fx^; *ChAT-Cre* for *Dlk-ΔMN*. (B) Schematic of the RiboTag approach. Expression of HA-tag Rpl22 in ChAT^+^ MNs made it possible to collect ribosome-associated mRNAs from the ventral horn of the spinal cord in both Ctl and *Dlk* KO MNs. (C) Principal-component analysis (PCA) plot shows grouping among replicates of each condition. The most variability is seen between injured and uninjured and then Ctl injured (copper circles) and *Dlk-ΔMN* injured (teal circles). (D) Heatmap of the first 30 most variable genes post SNC is shown. Most of the regeneration-associated genes (RAGs) are still upregulated in *Dlk-ΔMN*, although upregulation of some is diminished strongly (for example, *Coch,* *Npy*, and *Gal*). (E) Venn diagram of differentially expressed genes (DEGs). From 2,086 DEGs, a distinct subset (310) of the SNC-induced DEGs showed strong dependence on DLK (based on 1.5-fold change, an adjusted p value of less than 0.05). (F) Volcano plot comparing DEGs from injured MNs of *Dlk-ΔMN* vs. Ctl (injured) mice. For this plot, significant labeled DEGs are based on a fold change of 1, an adjusted p value of less than 0.05. (G) Bubble plot of Gene Ontology (GO) analysis in three categories: Biological Processes (BP), Cellular Component (CC), and Molecular Function (MF). The *Z* score suggests whether the genes in each significant GO term are likely to deplete (negative value) or increase (positive value). To calculate the *Z* score, the number of upregulated genes in each GO term is subtracted from the downregulated genes and divided by the square root of the total count of genes in that GO term..

To identify candidate mechanisms by which DLK activation in MNs could trigger microglial recruitment and synapse loss, we used RiboTag transgenic mice to capture MN-specific transcripts^42–44^ and to profile DLK-gated changes in gene expression at day 3 (D3) post SNC (Figures 3A and 3B). To perform these experiments, we generated Ctl (*Dlk*^+/+^;*ChAT-Cre;RiboTag*) and *Dlk-ΔMN* (*Dlk^fx/fx^;ChAT-Cre;RiboTag*) mice. Validating the approach, immunoprecipitation with a hemagglutinin (HA) antibody led to an enrichment for *Chat* transcript compared to the total input, while transcripts specific to other cell types (*CNPase* and *Calbindin*) were de-enriched (Figure S3A). *Dlk* transcript was enriched specifically in RiboTag-purified transcripts from Ctl animals and absent from *Dlk-ΔMN*; RiboTag mice, confirming DLK’s expression in MNs and its successful deletion with *ChAT-Cre* driver (Figure S3B). Principal-component analysis (PCA) confirmed dramatic differences between each experimental condition but not between experimental replicates (Figure 3C). The uninjured side of the spinal cord, which is used for most of the immunohistochemistry analysis in this study, was similar to naive animals (Figures S4A and S4B). Uninjured MNs from *Dlk-ΔMN* and Ctl mice showed some modest differences (Figure S4C).

Based on previous studies,^9,11,12,14,15^ we expected DLK signaling to be induced in injured MNs following SNC. We confirmed this by staining for phosphorylated c-Jun (p-c-Jun), a downstream target of DLK.^30,31^ p-c-Jun was dramatically induced in MNs of injured spinal cord in Ctl but not *Dlk-ΔMN* animals (Figures S5A–S5C). We were therefore most interested in genes that show DLK-dependent expression changes following SNC.

As expected from previous studies of axonal regeneration following PNI,^14,45,46^ SNC led to a strong induction of many regeneration-associated genes (RAGs). Based on the previously reported requirement for DLK in MN regeneration,^9^ along with its essential role in axonal regeneration in *Caenorhabditis elegans*,^11,12,47^ we were initially surprised that many of the RAGs were strongly induced by SNC in MNs deleted for *Dlk* (Figure 3D). These include the transcription factor ATF3, which plays a critical role in axonal regeneration^48^ and was used to identify axotomized MNs in Figures 1A, 1B, and 1D. In *Dlk-ΔMN* mice, MNs but not sensory neurons showed delayed regeneration at 3 days post SNC (Figures S6A–S6-D), indicating a cell-autonomous role for DLK in initiating regeneration. However, when assessed at later time points (21 and 50 days post SNC), MNs deleted for *Dlk* were capable of complete muscle reinnervation (Figures S6E and S6F). Therefore, DLK is not essential for axonal regeneration in the mammalian peripheral nervous system (PNS).

While SNC led to dramatic changes in many ribosome-associated transcripts, with 2,086 differentially expressed genes (DEGs), a distinct subset (310) of the SNC-induced DEGs displayed strong dependence on DLK (based on 1.5-fold change, adjusted p value of less than 0.05) (Figures 3E and 3F). Gene set enrichment analysis of the DLK-dependent SNC gene set (using both the Database for Annotation, Visualization and Integrated Discovery [DAVID] and the GeneSCF package) identified several significant functional classes of secreted proteins, including components of the immune system, neuropeptides. and cytokines (Figure 3G).

### DLK function in injured motor neurons triggers the induction of complement

**Figure 4.**
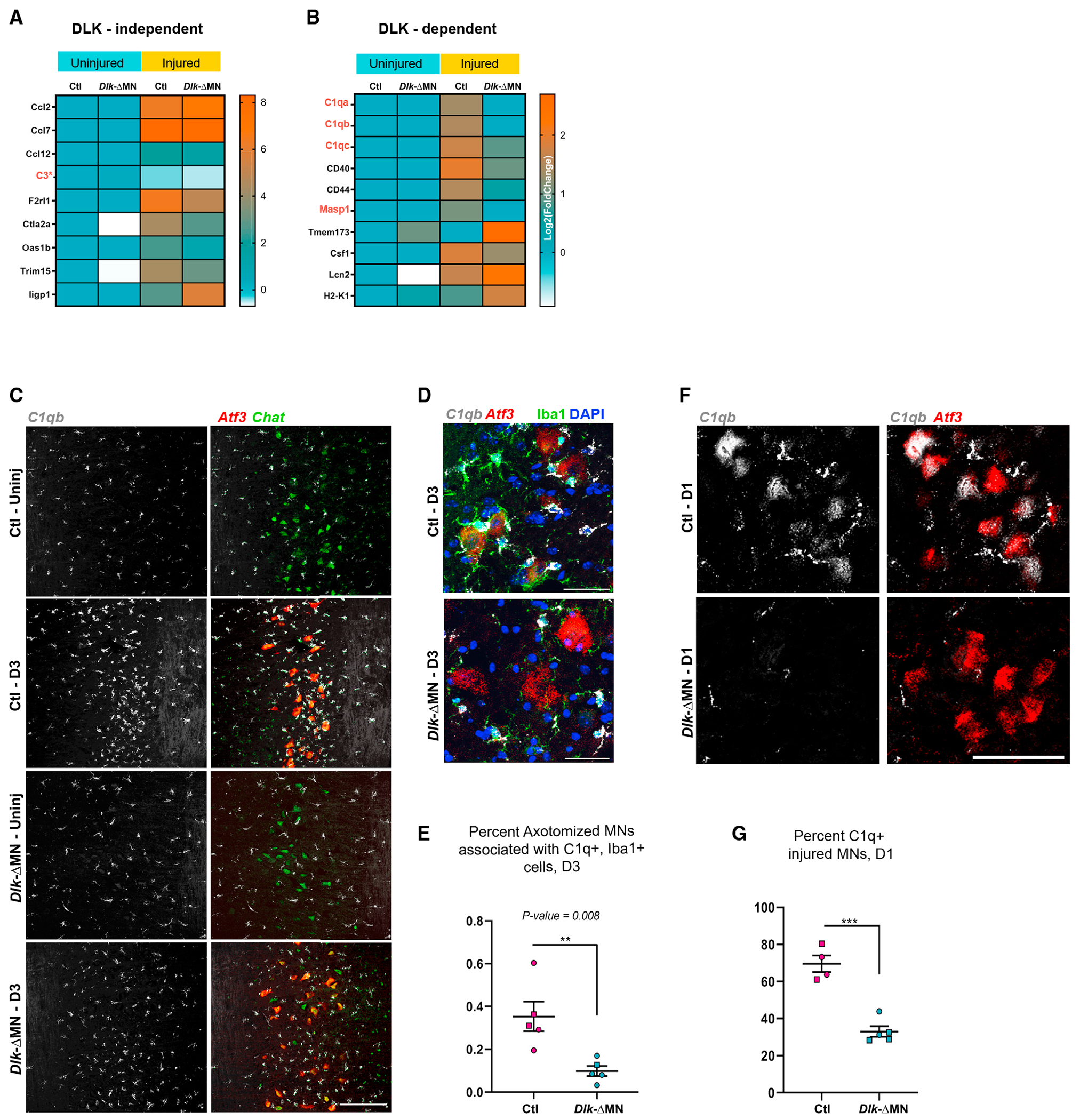
DLK function in injured motor neurons triggers *C1q* expression (A and B) Heatmaps of selected genes from the ribosome-associated transcripts in MNs associated with GO terms (innate immune response and immune response process). Cytokines such as CCL2 and 7 are not regulated by DLK. Members of the complement cascade *C1qa*, *C1qb*, *C1qc*, and *Masp1* show DLK-dependent induction in injured MNs. *Levels of *C3* do not show statistically significant changes; read counts <100. Genotypes are *Rpl22*^*HA/HA*^; *Dlk*^+/+^; *ChAT-Cre* for Ctls or *Rpl22*^HA/HA^; *Dlk*^fx/fx^; *ChAT-Cre* for *Dlk-ΔMN.* (C) *In situ* hybridization of *C1qb* (gray), *Chat* (green), and *Atf3* (red) 3 days post SNC. C1q is strongly expressed by non-neuronal cells that surround injured MNs, but this response is reduced in *Dlk-ΔMN* animals. Genotypes are *Rpl22*^HA/HA^; *Dlk*^fx/fx^; (*Cre* negative) for Ctls or *Rpl22*^HA/HA^; *Dlk*^fx/fx^; *ChAT-Cre* for *Dlk-ΔMN*. Scale bar, 200 μm. (D) Close-up of *in situ* hybridization of *C1qb* (gray), and *Atf3* (red) immunostained for Iba1 (green) and DAPI. Iba1 cells that express *C1qb* make extensive contacts with axotomized MNs in Ctl but not in *Dlk-ΔMN* animals. (E) Quantification of the number of Iba1^+^/C1q^+^ microglia associated with axotomized MNs (shown in D) in Ctl and *Dlk-ΔMN* animals. (F) *In situ* hybridization of *C1qb* (gray) and *Atf3* (red), 1 day post SNC, shows MN expression of *C1qb* in Ctl and not *Dlk-ΔMN* animals. Genotypes are *Rpl22*^HA/HA^; *Dlk*^fx/fx^; Cre negative for Ctls or *Rpl22*^HA/HA^; *Dlk*^fx/fx^; *ChAT-Cre* for *Dlk-ΔMN.* (G) Quantification of (F); percentage *C1qb*-positive axotomized MNs is calculated by counting the number of neurons that have *C1qb* puncta in them and dividing them by the total number of axotomized MNs.

Since the microglial response to axotomized MNs is strongly regulated by DLK, we focused our attention on immune system genes gated by DLK in injured MNs. While known neuronally expressed cytokines CCL2, CCL7, and Csf1 (previously proposed to be regulated by DLK in injured DRGs^22,23^) are not strongly regulated by DLK in injured MNs (Figure 4A), we noticed that all three subunits of C1q are induced in injured MNs in a DLK-dependent manner. In addition, Mannan-binding lectin serine protease 1 (MASP1), an alternative activator of complement in the C1q-independent lectin pathway, is also strongly induced by DLK in injured MNs (Figure 4B). The complement cascade is an important arm of innate immunity with known roles in directing the phagocytosis of synaptic components in development and disease.^49–52^ We therefore hypothesized that DLK signaling in injured MNs induces the activation of complement.

While complement components are normally highly expressed in glial cells, neuronal sources of complement are not well known. Therefore, we first examined the expression pattern of *C1q* in the spinal cord by *in situ* hybridization. At D3 post SNC, *C1q* expression is predominant in non-neuronal cells, particularly in the vicinity of injured MNs (Figures 4C and 4D). This enrichment of *C1q* expression in Iba1+ cells surrounding injured Atf3+ MNs was strongly reduced in *Dlk-ΔMN* mice (Figures 4D and 4E).

The RiboTag profiling demonstrated expression of *C1q* in MNs. Due to the strong expression of *C1q* in associated microglia, we could not confirm *C1q* expression in MNs at D3. However, at D1 post SNC, we observed compelling expression of *C1qb* in injured Atf3+ MNs. (Figures 4F and 4G). This early expression of *C1q* in axotomized MNs is diminished in *Dlk-ΔMN* animals, consistent with the RiboTag RNA sequencing (RNA-seq) dataset.

**Figure 5.**
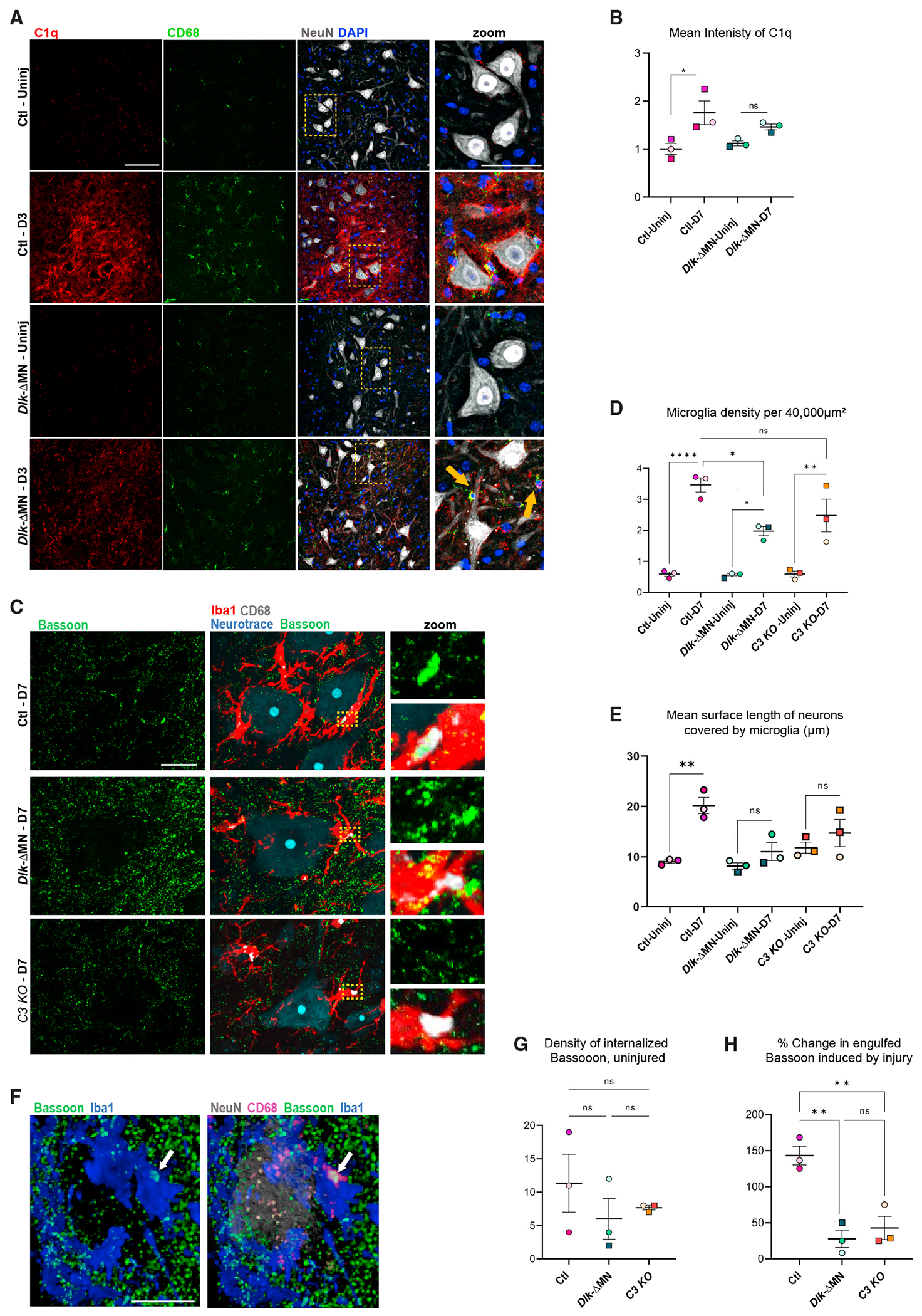
Neuronal DLK promotes C1q deposition and internalization of presynaptic components by Iba1^+^ cells (A) Immunostaining of C1q 3 days post SNC. Dotted boxes in the merged image are enlarged in the far right panel. Ctl axotomized MNs are heavily coated by C1q-positive puncta. However, in *Dlk-ΔMN* mice, C1q localization is largely restricted to CD68^+^ cells (indicated with yellow arrows). Genotypes are *Rpl22*^HA/HA^; *Dlk*^fx/fx^; (*Cre* negative) for Ctls or *Rpl22*^HA/HA^; *Dlk*^fx/fx^; *ChAT-Cre* for *Dlk-ΔMN.* (B) Quantification of mean C1q intensity surrounding MNs. A one-way ANOVA with the Tukey test for multiple comparisons was performed. *p < 0.05 (p = 0.0238). (C) Representative images of axotomized MNs in Ctl, *Dlk-ΔMN*, and *C3* KO 7 days post SNC. Insets show enlargements of CD68^+^ puncta that either contain or do not contain Bassoon. Scale bar, 20 μm. (D) Quantification of microglial density at D7 post SNC. (E) Extent of the motoneuron surface contacted by microglia at D7. From merged images of individual MNs, a line tool was used to measure length of contact interfaces between microglia and MNs (similar to measurements in Figure 2F). Statistical comparisons were evaluated for mean values per animal (n = 3 per genotype) using one-way ANOVA with the Tukey test for multiple comparisons. *p < 0.05, **p < 0.005, ***p < 0.005, and ****p < 0.0001. (F) 3D rendering of a Ctl neuron (Gray-NeuN) at D7 post SNC, which is surrounded by multiple Iba1^+^ cells (blue) and presynaptic Bassoon puncta (green). The arrow points to an example of an object that is positive for Bassoon that colocalizes within a CD68^+^ monocyte lysosome. Scale bar, 20 μm. (G) Quantification of the density of engulfed Bassoon puncta, which are colocalized with CD68 within Iba1^+^ cells. This analysis was done for the entire pool of MNs in L3–L6 lumbar segments imaged from three or four longitudinal sections, n = 3 mice per genotype. A one-way ANOVA with the Tukey test for multiple comparisons was performed. ****p < 0.0001. (H) The percentage change in engulfed Bassoon at D7 post SNC was measured per animal, comparing injured (IL) to uninjured (CL) regions in the same longitudinal sections described for (G).

We then examined C1q protein by antibody staining (Figures 5A and 5B). C1q staining is only sparsely detected in CL uninjured MNs; however, a dense net of C1q surrounds Ctl injured MNs at D3 post SNC. This induction of MN-associated C1q in response to injury was reduced in *Dlk-ΔMN* tissues (Figures 5A and 5B). Furthermore the C1q staining in injured *Dlk-ΔMN* tissues did not appear extracellular, but instead was concentrated within CD68^+^ cells (arrows in Figure 5A). From these data, we infer that DLK signaling in injured MNs triggers cellular events that lead to the extracellular accumulation (and likely opsonization) of C1q.

A previous study reported that the loss of synaptophysin from axotomized MNs still occurred in *C1q* KO mice but was impaired in *C3* KO mice.^34^ Since the classical C1q-dependent pathway is one of several mechanisms that activate C3 (and another is the lectin pathway, which may also be induced by DLK), we turned to C3 as an essential component of the complement pathway and examined *C3* KO animals. *C3* KO mice showed phenotypes that resembled *Dlk-ΔMN* mice but were more mild (Figures 5C–5H). Microglial density increased surrounding injured MNs similarly to wild-type mice (Figure 5D); however, these microglia showed a mild reduction in their extent of contact with the injured MN (Figures 5C and 5E). We were unable to detect an induction of C3 protein or transcript and noted that its expression levels are low in the adult spinal cord (Figures 4A and S7). Together, these observations suggest that the complement pathway participates in the microglial response to axotomy downstream of DLK activation but may not be the sole mediator of DLK-gated responses.

### Microglia internalize presynaptic components from axotomized MNs

Complement is previously known for its role in directing the phagocytosis of synaptic components by microglia. However, in contrast to developmental pruning, prior studies of synaptic stripping from axotomized MNs have not detected evidence for microglia internalizing synaptic components and have instead proposed alternative relationships of microglia with injured MNs.^1,3,53^ We noticed the presence of the presynaptic marker Bassoon within CD68^+^ structures within Iba1+ cells, indicating they were internalized by microglia and localized inside lysosomes (Figures 5C and 5F). The density of internalized Bassoon showed variability among animals of all genotypes (Figure 5G) but increased significantly around injured MNs in Ctl animals (D7) post SNC (Figures 5C and 5H). In *Dlk-ΔMN* and *C3* KO animals, the density of internalized Bassoon was not strongly induced following injury (Figure 5H). Together, these observations suggest that cellular components of the presynaptic inputs onto axotomized MNs are phagocytosed by microglia, and this process is triggered by events in MNs regulated by DLK (Figure S8).

## DISCUSSION

### Role of neuronal signaling and microglia in axotomy-driven synapse loss

First described in the 1960s, the stripping of synaptic inputs from axotomized MNs is one of the oldest forms of microglial-assisted neuronal plasticity^2^; however, the mechanism is still poorly understood.^1^ Phagocytosis of synapses by microglia has not been documented for axotomized motoneurons. Instead, electron micrographs showing the interdigitation of microglial processes between axotomized motoneurons and their presynaptic boutons led to the proposal that microglia act as a wedge to lift boutons away from the cell body.^2,54^ Treatments that impair microglial proliferation, combined with comparative studies noting a lack of correlation between microglial density and synapse loss,^4–6^ have led to the prevailing view that axotomy-driven synapse loss is driven by cell-intrinsic changes triggered within the injured neuron. However, the actual events that occur within the injured motoneuron that lead to synapse loss are not known.

A handful of studies have noted that axotomized motoneurons show reduced expression levels of certain synaptic adhesion proteins (including neuroligins and SynCAM1 as detected by *in situ* hybridization).^55,56^ However, whether a reduction in synaptic protein levels is sufficient to drive synapse loss is not clear. Our discovery that the DLK kinase, which becomes activated specifically in axotomized neurons,^9,10^ functions as upstream regulator of synapse loss now opens a window into studying the mechanism of this form of structural plasticity.

While the expression levels of some synaptic proteins are gated by DLK in axotomized neurons (Figure S9), some of the strongest DLK-regulated targets include neuropeptides and innate immunity genes. This brings the previously debated role of microglia back into the picture. We found that DLK signaling induction in injured neurons is required for the recruitment of Iba1^+^, CD68^+^ cells to form close interactions with the injured neuron, and internalize the presynaptic cytomatrix components Bassoon. These data suggest an active role for microglia in the removal and/or clearance of synaptic inputs from axotomized neurons. It is also noteworthy that the clearance occurs to presynaptic components of neurons that are not directly injured by the PNI. How this is achieved downstream of DLK signaling activation is an important future question.

### DLK directs the activation of complement and inflammation

Our RiboTag data indicate that DLK induces the expression of all three subunits of C1q. In addition, DLK triggers expression of Mannan-binding lectin serine protease 1 (MASP-1), a parallel upstream activator of complement. A previous study showed that synapse loss still occurs in *C1q* KO mice but is reduced in *C3* KO mice.^34^ The co-induction of MASP-1 together with C1q suggests that both classical and lectin arms may work in parallel to activate complement downstream of DLK.

We note that neuronal DLK is required for the induction of C1q expression in both neurons and microglia. Because C1q alone is not required for synaptic stripping,^34^ the role of its early expression by neurons is not clear. However, regardless of the cellular source, the induced expression of C1q (and MASP-1) alone does not provide a sufficient explanation for how either the classical or lectin arms of complement become activated. MASP-1 is synthesized as a zymogen that is inactive on its own; its activation is triggered through assembly into a complex of proteins with pathogen recognition molecules of the lectin pathway. Similarly, C1q circulates in an inactive state; a number of ligands can lead to its activation and opsonization, although its best-known trigger in the nervous system is binding to phosphatidylserine (PS) on the surface of apoptotic cells and debris.^57^ However, following injury of the peripheral nerve, there is no direct physical damage in the spinal cord. We therefore hypothesize that an additional event (or set of events) must occur, in parallel to the induction of C1q and MASP-1 expression levels, to trigger activation of the microglial responses, and that this additional event is also gated by DLK signaling in injured neurons.

In contrast to C1q and MASP-1, we did not observe an induction of *C3* transcript in the injured spinal cord (Figure 4A). C3 cleavage (rather than transcript induction) is a critical step in the activation of the complement pathway, and the C3 cleavage product, C3b, functions to tag its opsonized targets for phagocytosis. We therefore attempted to visualize C3b using antibodies that have been used to show C3 localization at synapses that are lost during developmental pruning, aging, and neurodegenerative disease.^58–61^ However, we failed to detect specific localization (Figure S7A) and note that previous studies, to our knowledge, have not shown synaptic C3 staining validated against *C3* KO tissue. While the genetic data suggest that C3 plays a role in axotomy-driven synapse loss,^34^ we acknowledge that direct detection of C3 activation could not be confirmed. We also note that the microglial responses to injury in *C3* KO mice did not fully recapitulate the phenotypes of *Dlk-ΔMN* (Figure 5D). It is likely that additional pathways downstream of DLK participate in the activation of microglia and loss of synapses.

Microglia-associated synapse loss has been described in many other contexts, including developmental pruning and models of neurodegenerative disease.^49^ Deeper study of axotomy-driven synapse loss and the role of DLK may bring future insights into the cellular mechanisms of synapse loss in these other contexts.

### A new role for DLK

Inhibition of DLK has been shown to rescue microglial activation in a SOD1-G93A mouse model of ALS^24^ and dorsal horn inflammation in spared nerve injury (SNI) and sciatic nerve transection (SNT) models of chronic pain.^22,23^ However, previous studies attributed DLK’s role in inflammation to the regulation of cytokines (Csf-1 and CCL2), which we observed are still induced in *Dlk* KO motoneurons. While DLK-dependent induction of C1q subunits and MASP-1 was not previously noted, published datasets suggest that these genes are also gated by DLK in dorsal root ganglion (DRG) neurons.^14^ It is therefore an attractive hypothesis that DLK gates the induction of complement from other neuron types, which is an addressable question for future studies.

DLK’s orthologue was originally discovered in *C. elegans* screens for its essential role in enabling damaged axons to regenerate.^11,12^ It was therefore initially surprising that regeneration-associated genes (RAGs) are still robustly induced in motoneurons deleted for DLK. However, our findings are compatible with recent studies in zebrafish and mice.^62,63^ These studies have documented robust axonal regeneration in *Dlk* KO animals and an intersecting role for DLK with a related kinase LZK.^62,63^ Therefore, while DLK appears to influence axonal regeneration in some cell types,^7,14^ it is not globally required for axonal regeneration in the mammalian nervous system.

Our current study points to a new role for DLK in regulating synapse loss, which may be independent of its other roles in axonal regeneration and neuronal death. On face value, this role is counter-intuitive to the original discovery that its *Drosophila* homolog, Wallenda, can mediate nerve terminal overgrowth at the *Drosophila* neuromuscular junction (NMJ).^10^ However, later studies have shown that Wallenda signaling activation actually induces a reduction in the number of postsynaptic densities opposed by presynaptic release machinery.^64,65^ Whether these synaptic changes involve contributions by additional cell types has not been previously considered. Our current data suggest that DLK may regulate postsynaptic mechanisms within the injured MN. We note that previous studies have identified roles for Wallenda in regulating dendritic branches and cytoskeletal dynamics in *Drosophila* sensory neurons.^66,67^ In addition, a study in mouse models of Alzheimer’s disease noted a potential role for DLK in regulating dendritic spine morphology,^24^ and DLK has been observed to interact biochemically with the postsynaptic scaffolding protein PSD-95.^19^ Finally, a longitudinal study in cultured cortical neurons noted that treatment with DLK inhibitors prolonged synaptic connectivity *in vitro*.^68^ We consider it likely that DLK signaling activation induces cell-intrinsic changes that affect synapses, which may work in concert with complement and microglial responses to mediate synaptic structural plasticity (Figure S8).

A very recent study has described microglial-mediated loss of synapses in the dorsal horn in a mouse model of neuropathic pain.^69^ Knowing that DLK contributes to injury-induced allodynia,^23^ whether it directs some of these synaptic changes is an interesting future question. The induction of synaptic loss and inflammatory responses may be grouped with other known responses gated by DLK into an overarching theme of neuronal plasticity. Axonal regrowth, synapse loss, and neuronal death are all distinct but equally impactful mechanisms for structural plasticity used by the nervous system to adapt to situations that impair neuronal circuits. As a stress response pathway induced by nerve injury and other major neuronal impairments, DLK’s evolutionarily conserved role may be to serve as a gatekeeper of plasticity mechanisms.

### Limitations of the study

Since the *ChAT*-Cre driver removed *Dlk* from motoneurons during development, it is possible that the defective responses to injury are the result of an aberrant developmental event. It is also theoretically possible that the RiboTag homozygous background synergizes with *Dlk* KO to reveal the synapse loss defects. We note that a prior study showed that microgliosis in the spinal cord could be prevented by DLK inhibitors following sciatic nerve injury,^23^ so the microglial response, if not synapse loss per se, appears to depend upon DLK signaling activation following injury.

We also do not know the full extent of synapse loss that depends upon DLK. Axotomized MNs still retain many synapses, and what is retained versus lost appears to vary in different injury paradigms and organisms (reviewed in Alvarez et al.^1^). It is also likely that different synapse types are lost via different mechanisms. For instance, it has been shown that the permanent removal of VGlut1 inputs from proprioceptor 1a neurons is controlled by CCR2 signaling; however, bulk stripping does not require CCR2.^4^ Another intriguing previous study has suggested a requirement for MHC1 in selective maintenance of inhibitory synapses.^33^ We have not yet evaluated DLK’s role in all synapse types. We note that synaptophysin, which is expected to be present at all synapses, can show differences in different strain backgrounds, so care must be taken when comparing synapse loss across genetic conditions, especially for measurements that rely on intensity rather than bouton counting. It also remains to be determined what happens to the postsynaptic densities and whether DLK signaling affects synapses independently of the responses in microglia.

We did not confirm the localization of C1q or other complement components to synapses per se. Compared to other studies in the developing CNS,^49,58,70,71^ the distribution of C1q in the ventral horn following SNC appears more broadly distributed and could include localization to non-synaptic structures. The full chain of events that underlie complement activation and its downstream consequences following PNI remain to be determined.

## STAR*METHODS

### RESOURCE AVAILABILITY

#### Lead contact

Further information and requests for resources and reagents should be directed to and will be fulfilled by the lead contact, Catherine Collins, cxc1215@case.edu.

#### Materials availability

This study did not generate new unique reagents.

#### Data and code availability

##### Data:

RNA-seq data have been deposited at GEO (GSE213987) and are publicly available as of January 22, 2024. Accession numbers are listed in the key resources table. Microscopy data reported in this paper will be shared by the lead contact upon request.

##### Code:

This paper does not report original code.

Any additional information required to reanalyze the data reported in this paper is available from the lead contact upon request.

### EXPERIMENTAL MODEL AND STUDY PARTICIPANT DETAILS

#### Animals

All procedures involving mice were performed in accordance with guidelines developed by the National Institutes of Health and were approved by the Institutional Animal Care and Use Committee (IACUC) at the University of Michigan and Case Western Reserve University. Animals were housed in single sex groups of 3–5 per cage in standard ventilated cage housing in a room with controlled temperature (22 ± 1C), humidity (55%) and 12h light/dark cycle with *ad libitum* access to water and food. Adult (12–15-Week-old) males and females on a C57BL/6 background from the following mouse lines were used. Sex is tracked in the graphs shown (circles are female, squares are male). The genotypes used for each figure are described in the figure legend.

We used the following mouse lines:

*Dlk conditional KO: Dlk*^fx/fx^ (Miller et al. 2009)

RiboTag: *Rpl22^HA^*: B6N.129-Rpl122tm1.1Psam/J (JAX Stock #011029)

*Chat^Cre^*: *ChAT-IRES-Cre*: B6; 129S6-*ChAT*tm(Cre)/Lowl/J (JAX stock #006410)

Rosa-tdTomato line (JAX stock # 007914 (Ai14))

Tmem119-2A-EGFP reporter mouse line (JAX stock # 031823)

*C3 KO:* B6.129S4-C3tm1Crr/J (JAX stock #029661)

Ribosomal profiling from MNs was achieved by crossing *Rpl22^HA/HA^* (RiboTag) and *ChAT^Cre^* mice, then subsequently crossed to *Dlk* conditional mice to generate *Rpl22*^HA/HA^;*Dlk^fx/fx^;ChAT*^Cre/+^ (*Dlk-ΔMN*) experimental mice and *Rpl22^HA/HA^*;*Dlk^fx/fx^*;*ChAT*^+/+^ or *Rpl22^HA/HA^*;*Dlk*^+/+^;*ChAT*^Cre/+^ control mice for histological and RiboTag/Sequencing experiments, respectively. (All mice were homozygous for RiboTag (*Rpl22*^HA/HA^)).

For stripping studies and to be able to mark the perimeter of the motor neurons, the Rosa-TdTomato line Ai14 was crossed into the background of *Rpl22^HA/HA^*;*Dlk^fx/fx^*;*ChAT*^Cre/+^ mice to generate Ai14/Ai14 *Rpl22^HA/HA^; Dlk*^+/*fx*^; *ChAT*^Cre/+^ (Tdtomato; *Dlk-ΔMN*). These mice were crossed with *Rpl22^HA/HA^; Dlk*^*fx*/+^ to generate *DlkΔMN* and control littermates for comparison. (*DlkΔMN* = Ai14/14; *Rpl22^HA/HA^; Dlk*^fx/fx^; *ChAT*^Cre/+^ and Ctl = Ai14/14; *Rpl22^HA/HA^; Dlk*^+/+^; *ChAT*^Cre/+^*)*

For microglial inhibition using Csf1r inhibitor, PLX5622, Tmem119^eGFP/eGFP^ (Tmem119^eGFP^) mice were used as an endogenous microglia reporter. Tmem119^eGFP^ was crossed into the *Dlk^fx/fx^*;*Chat*^Cre^ background to generate *Tmem119*^eGFP/eGFP^;*Dlk^fx/fx^*; *ChAT*^Cre/+^ (Tmem119^eGFP^;*Dlk-ΔMN*) experimental mice. These were compared to *Tmem119*^eGFP/eGFP^;*Dlk^fx/fx^*; Cre-negative sibling controls.

### METHOD DETAILS

#### Surgical procedures

Mice were anesthetized with 2–3% Isoflurane mixed with Oxygen. Carprofen was administered subcutaneously as an analgesic 15 min before performing the surgery and then once a day for 48 h post-surgery. At the level of mid-thigh, a 1 cm incision was made through the skin, and muscle and sciatic nerve were exposed. Using fine forceps (#11399-80, Fine Science Tools), the sciatic nerve was crushed for 30 s. Sutures were used to close the muscles and to close the skin, and clips (Roboz, RS-9258) were used. Naive mice received no injury.

#### MICROGLIAL DEPLETION WITH PLX5622

A chow diet containing the Csf1r inhibitor PLX5622 (obtained from Plexxikon) was produced by Research Diets Incorporated with 1200 mg of active form of PLX5622 per kg of the diet. Mice were fed the formulated chow (estimated dosage of 1200 ppm) starting 2 weeks prior to SNC and continued until the mice were sacrificed. Control mice were kept on a diet containing the same base formula (AIN-76A formula, irradiated, from Research Diets).

#### Tissue preparation and immunohistochemistry

Mice were perfused transcardially with Phosphate buffer saline (PBS), then the entire spinal cord was isolated by the hydraulic extrusion (Kennedy et al., 2013). The lumbar segment was then divided at the midline to separate uninjured (contralateral) and injured (ipsilateral) sides before flash-freezing the samples in liquid nitrogen. Samples were similarly collected from naive mice. For degeneration and reinnervation analysis, the sciatic nerve and extensor digitorum longus (EDL) muscles were collected, respectively. The EDL was fixed for 20 min at room temperature using 4% paraformaldehyde (PFA), while spinal cords and sciatic nerves were fixed with 4% PFA overnight. Samples were washed 3 times with 1X Phosphate buffer Saline (PBS) after fixation and transferred to 30% sucrose and kept at 4°C. Following sucrose saturation, samples were embedded in O.C.T. Compound (Fisher, 4585). For histochemical purposes tissues were cryosectioned (Leica 3050S) to obtain longitudinal serial sections of different thicknesses (spinal cord: 20 μm, sciatic nerves: 15 μm; EDL: 50 μm). Spinal cord sections were collected on 3 alternating slides, 6 sections per slide, such that the entire motorneuron pool in L3-L6 could be sampled with 4–5 longitudinal sections on two of the 3 slides.

For immunohistochemistry, samples were washed with 1X PBS to remove the OCT for 5 min, then permeabilized for 30 min using 0.1% Triton X-100. Immunostaining of spinal cord sections was carried out on the surface of a coated slide within a ring drawn by an ImmEdge hydrophobic pen (Vector Biosciences). Blocking was carried out for 30 min to 1 h at room temperature in 1X PBS containing 0.1% Triton X-, 10% donkey serum (Jackson ImmunoResearch), and M.O.M blocking reagent (Vector Laboratories). Sections were then incubated with primary antibodies dissolved in 1X PBS containing 0.1% Triton X-, and 5% donkey serum/goat serum. For Choline Acetyltransferase (ChAT) staining, antigen retrieval step (10 mM Sodium Citrate pH:6 at 95°C for 3 min) was carried out prior to permeabilization. Primary antibodies used: goat anti-ChAT (Sigma, 1:100), Rabbit anti-HA (Cell Signaling, 1:100), Mouse anti-NeuN (Sigma, 1:500), Rabbit anti-*c*-Jun Serine 73 phosphorylated (Cell signaling, 1:100), Rabbit anti-STMN2/SCG10 (Novus Biologicals, 1:100), mouse anti-beta-Tubulin III (Sigma, 1:200). Secondary antibodies used: 543 Donkey anti Rabbit, 488 Donkey anti-Mouse, 647 Donkey anti-goat (all from Jackson Laboratories) along with conjugated α-Bungarotoxin CF543A (Biotium, 1:75).

#### Immunoprecipitation and RNA isolation

Homogenization and immunoprecipitation (IP) of Rpl22-HA-tagged ribosomes was performed according to published protocols (Sanz et al., 2009) with some modifications.^42^ Briefly, spinal cord tissue as described above was separated at the midline and flash frozen in liquid nitrogen. Tissue was homogenized with a glass Dounce homogenizer with tight-fitting pestle in freshly made homogenization buffer (50 mM Tris, pH 7.4, 100 mM KCl, 12 mM MgCl2, 1% Nonidet P-40 [NP-40] supplemented with 200 U/mL RNasin (Promega), 1 mg/mL heparin, 100 μg/mL cycloheximide, and protease inhibitor mixture (Sigma-Aldrich). Samples were incubated at 4°C with gentle rotation for 20–30 min, followed by centrifugation at 10,000 Xg at 4°C for 10 min. The supernatant was collected and a small fraction of the input sample was saved. The remaining sample was then precleared at 4°C for an hour with Protein G magnetic beads (New England Biolabs) with constant rotation. After separating supernatants from the magnetic beads using a magnetic rack (Invitrogen), samples were toppled at 4°C for 4 h with HA antibody (Biolegend, MMS-101P, 1:150 dilution). After the 4-h preincubation, Protein G magnetic beads were added and samples were left to incubate under constant rotation at 4°C overnight. The next day, supernatants were separated from the magnetic beads. The beads associated with immunoprecipitated ribosomes were then washed in a high salt buffer containing 50 mM Tris, pH 7.4, 300 mM KCl, 12 mM MgCl2, 1% NP-40 supplemented with 100 μg/mL cycloheximide 3 times. After the third wash, the beads were eluted via a 30 s vortex in the lysis buffer (RNeasy RLT buffer, and Beta Mercaptoethanol or BME). RNA was then isolated from the resulting supernatant/eluate using RNeasy Plus Micro Kit (Qiagen) per manufacturer instructions.

#### RNAscope in *situ* hybridization

*In situ* hybridization was performed on fixed frozen samples to analyze multiple targets using the RNAscope Multiplex Fluorescent v2 Assay according to the manufacturer’s instructions. We used a probe mix that combined diluted C2 and C3 probes into a solution that contained 1x of C1 probe. For amplification A1, A2, and A3 solutions were applied for 30, 30, and 15 min, respectively. Each probe was then developed sequentially starting with an HRP-C1 (15 min), TSA secondary (30 min),and HRP Blocker (15 min). All developing steps were then repeated for C2 and C3 probes. The TSA secondaries (NEL741001KT, NEL745001KT, NEL744001KT; Akoya Biosciences) in these experiments were used at a concentration determined independently for each probe. Following the last wash, samples were mounted with DAPI Fluoromount-G (SouthernBiotech, 0100-20) and imaged within 3–5 days.

#### RT-qPCR

RNA concentration was measured using a Nanodrop spectrophotometer and/or a 2100 Bioanalyzer Instrument (Agilent Genomics). The remainder of the sample was used for cDNA preparation with Superscript III First-Strand Synthesis SuperMix (Invitrogen). RT-qPCR was performed using primer sets in Table S1, FastStart Universal SYBR Green Master Mix (Roche), and a 7900HT Fast Real-Time PCR System (Applied Biosystems). For each sample, we had 3 technical replicates and 2–3 biological replicates. We averaged the resulting CT of our 3 technical replicates per sample (biological replicate). The result was log2 transformed and then relative expression was graphed compared to input for each gene.

#### RNAseq library preparation and analysis

RNA sequencing was performed on 3 animals per condition, all males. The conditions included: completely naive animals, and both ipsilateral (injured) and contra late ral (uninjured) sides of the spinal cord from D3 post SNC, from control (*Rpl22*^HA/HA^; *Dlk*^+/+^; *Chat*^Cre/+^) and *Dlk-ΔMN (Rpl22*^HA/HA^; *Dlk*^fx/fx^; *Chat*^Cre/+^) conditional KO mice. After the RiboTag IP (described above) Isolated RNAs (RNA integrity value (RIN) > 7.5) were sent to the University of Michigan Sequencing Core for library prep using “SMART-Seq v4 Ultra Low Input RNA Kit for Sequencing”. The core used the Covaris shearing and ThruPlex library prep, along with PolyA selection to generate cDNAs. Paired end 50 bp sequencing was performed on a HiSeq4000 platform. Fastq files received from the core were quality-checked using FastQC, then adapter trimming was performed using Illumina Thruseq adaptors and BBDuk (“BBMap” n.d.). Trimmed reads were then mapped to the mouse genome (GRCm38.p6) using the STAR/2.5.2a aligner.^72^ We performed post mapping quality control using QoRTs.^74^ One of the *Dlk-ΔMN* naive samples behaved differently from other samples and showed bias towards its 3′ end so it was removed from our downstream analysis. DESeq2 package was used in R for differential expression analysis.^75^ Multiple differential expression analysis was performed using either control naive or control uninjured as the reference point. Heatmaps were generated using the ggplot2 package.^76^ Volcano plots were generated using the EnhancedVolcano package.^77^ For gene set enrichment analysis and pathway analysis, we used the DAVID platform (“DAVID Functional Annotation Bioinformatics Microarray Analysis” n.d.) and the GeneSCF in bash script.^78^ For DAVID we used all the genes that were identified in our sequencing as a background input. To plot the gene sets a combination of ggplot2^76^ and GoPlot^79^ packages were used.

### QUANTIFICATION AND STATISTICAL ANALYSIS

#### Statistical analysis

For statistical analysis in all our figures except Figures 4 and S4, we used GraphPad Prism software. Since males and females were combined in most experiments, we indicated male animals with square symbols and females with circles to track the sex. All plots are presented as Mean ∓ SEM. For some figures we used a Superplot format^80^ to show individual neuron measurements for each animal. Two types of statistical analysis were done; in each case the method used is indicated in the Figure legend. For comparing the means per animal, a paired t test was used between the injured and uninjured conditions per genotype with 95% confidence interval and * significance is considered p value less than 0.05, ** <0.005 and *** <0.0005. For other quantifications, A One-Way ANOVA with the Tukey test for multiple comparisons was performed. * 0.05, ** <0.005 and *** <0.0005 **** is p Value <0.0001.

For Figures 4 and S4, we used R to run the statistical analysis. Details of the analysis can be found in the method section. For statistical analysis, P-Values were initially measured using the DESeq2 package (Love, Anders, and Huber 2014). Then using dplyr package,^81^ we performed multiple types of correction analysis to adjust for P-value. Eventually, q-values generated by Benjamini-Hochberg method were used for selection of differentially expressed genes and downstream analysis. Our QC analysis showed trends of possible mRNA degradation in one of our Dlk-ΔMN naive samples, so we omitted this sample from downstream analysis. All animals used for the RNAseq experiments and mentioned figures were males.

#### Quantification of immunohistochemical and in *situ* hybridization analysis: *Regeneration analysis*

To recognize the site of injury we used a few criteria. First, forceps used to perform the nerve crush were coated in charcoal to mark the site of the injury. Second, after the fixation and sucrose saturation, we shortened the nerve proximal to the injury to facilitate finding the injury site after sectioning. Third, we binned IHC images of the entire nerve into sections of 50 μm width. The bin that contained the highest mean intensity for SCG10 was identified to be the site of injury. To obtain the ‘regeneration index’ we measured the distance between the bin of highest SCG10 intensity (the injury site) to the point at which it dropped to 50% of the maximum SCG10 intensity, as previously described in (Shin et al. 2012)^9^. To measure MN axonal regeneration, we used IHC images of entire nerves costained with ChAT and SCG10. We measured the distance between the tip of the farthest-extending ChAT positive axon and the injury site. ***Reinnervation analysis:*** Innervation analysis was performed on EDL longitudinal muscle sections, at D21 and D50 post SNC. The reinnervating nerve was visualized by staining for synapsin and then innervation status of each BTX+ NMJ was scored from 0 to 5, with a score of 0 indicating complete denervation and a score of 5 indicating full reinnervation.^82^ ***phospho-c-Jun quantification:*** DAPI was used to define the regions of interest round nuclei of motor neurons in the L3-L6 level at the injured (IL) and uninjured (CL) side of the spinal cord. Mean pixel intensity for p-*c*-Jun was measured at D1 post SNC. At D3 post SNC, the number of p-*c*-Jun positive nuclei in the injured side of the spinal cord was counted for both control and *Dlk-ΔMN*. This number was divided by the total number of MNs (marked by ChAT) to obtain the percentage. ***Quantification of synaptic markers:*** All quantifications were carried out with Volocity software while blinded to the genotype and conditions. ***Synaptophysin:*** We measured the density of synaptophysin staining that surrounded individual MNs. Injured MNs were identified based on location (ipsilateral side) and ATF3 expression. We used ChAT-Cre; Ai14 (Rosa-TdTomato) mice to delineate the surface of MNs. This outline of the MN cell body was traced using a freehand tool and defined as a region of interest. The total synaptophysin intensity within this area was divided by its surface area to estimate mean intensity of synaptophysin per MN. For each section, the mean intensity of synaptophysin in the uninjured (CL) area was used for normalization. For the injured side 100–150 neurons were counted per animal and for the uninjured 50–80 neurons were used for analysis. The mean intensity was measured by calculating the average of mean pixel intensity of all neurons on the injured or uninjured side of the spinal cord. ***Bassoon:*** Given that Bassoon specifically coated the perimeter of the motor neuron cell body, we quantified it differently from synaptophysin. The region of interest for intensity measurements was defined first by tracing the cell body outline. The dilate option in Volocity software was then used to make a second region of interest expanded 20 μm from the first in all directions. The donut-shaped area in the middle was used for intensity measurements. The sum intensity was divided by the donut area to calculate the mean density of Bassoon per MN. ***VGlut1 and VAChT:*** These synapses have individual puncta/boutons around the motor neuron cell body and on primary dendrites. The surface of each MN, based on TdTomato, was traced using the freehand tool to define the region of interest. Within this region the ‘find object’ tool was used in Volocity to find and count each VGlut1 and VAChT bouton. The total count was then normalized to the perimeter for the region of interest per MN to measure the density of VGlut1/VAChT synapses per 100 μm. ***Percent of Field covered by microglia*** (for Figure 2B): the fraction of pixels that were Iba1^+^ within 780 × 780 μm^2^ field of view surrounding MNs was measured for 4–5 longitudinal sections that covered the L4-L6 motoneuron pool from contralateral (uninjured) and ipsilateral (injured) sides. ***Microglial density and CD68 quantification*** (for Figures 5 and S2): The ‘Find Object’ tool in Volocity was used to identify and count Iba1^+^ cells (or Tmem119-GFP^+^ cells, for Figure S2) within the imaged areas. The imaged areas covered the ventral horn of L4-6 segments, in the areas containing MNs in both the injured (contralateral) and uninjured (ipsilateral) side. A similar method was done for CD68^+^ puncta for Figure 2C. ***Percent of MN surface contacted by microglia:*** the line function in Volocity was used to measure the perimeter of individual MNs based on NeuN staining, and then separately to measure the segments of the perimeter that were in contact with Iba1^+^ cells. ***C1q In situ hybridization and immunostaining quantification:*** For RNAscope *in situ* hybridization analysis, we counted the number of injured neurons (using *Atf3*) that contained any *C1q* puncta. This number was then divided by the total number of MNs counted and presented as the percent of injured MNs with *C1q* expression. For D3 post SNC, quantification of C1q immunostaining was performed by tracing around each motor neuron and finding the mean pixel intensity of C1q in the area selected around each MN. ***Quantification of bassoon internalization:*** Volocity software was used to identify Bassoon puncta that colocalize with CD68^+^ within Iba1^+^ cells, and then manually checked while blinded to genotype/condition. All puncta were counted within 40,000 μm^2^ images that covered the entire MN pool in L4-L6 segments.

## Supplementary Material

1

## Figures and Tables

**Table:** KEY RESOURCES TABLE

REAGENT or RESOURCE	SOURCE	IDENTIFIER
Antibodies		
Goat anti-Choline Acetyltransferase	Millipore/Sigma	(Millipore Cat# AB144P, RRID:AB_2079751)
Rabbit monoclonal anti-HA-tag (C29F4) - For Western Blot	Cell Signaling Technology	(Cell Signaling Technology Cat# 3724, RRID:AB_1549585)
Purified anti-HA.11 Epitope Tag Antibody (for RiboTag IP)	Biolegend	(BioLegend Cat# 901503, RRID:AB_2565005)
mouse monoclonal anti-NeuN (A60)	Millipore	(Millipore Cat# MAB377, RRID:AB_2298772)
Rabbit anti-phospho-*c*-Jun (Ser 73) monoclonal (D47G9)	Cell Signaling Technology	(Cell Signaling Technology Cat# 3270, RRID:AB_2129575)
Rabbit polyclonal anti-STMN2/SCG10	Novus	(Novus Cat# NBP1-49461, RRID:AB_10011569)
mouse anti-beta-Tubulin III monoclonal (clone 5H16)	Sigma-Aldrich	(Sigma-Aldrich Cat# T8578, RRID:AB_1841228)
Rabbit anti-ATF3 (polyclonal)	Novus	(Novus Cat# NBP1-85816, RRID:AB_11014863)
Rabbit anti-Iba1(polyclonal)	Wako	(FUJIFILM Wako Shibayagi Cat# 019–19741, RRID:AB_839504)
Rat anti-mouse CD68 monoclonal (clone FA-11)	Bio-Rad	(Bio-Rad Cat# MCA1957, RRID:AB_322219)
Rabbit anti-mouse c1q monoclonal (clone 4.8)	Abcam	(Abcam Cat# ab182451), RRID:AB_2732849)
Rabbit Monoclonal anti-C3 [EPR19394]	Abcam	(Abcam Cat# ab200999) RRID:AB_2924273
Gt anti Rat Complement C3	MP Biomedicals	MP Biomedicals (SKU:0855730)
Rat monoclonal anti-C3 [11H9]	Abcam	(Abcam Cat #ab11862) RRID:AB_10288263
Rabbit polyclonal anti-C3	Abcam	(Abcam Cat# ab11887) RRID:AB_298669
Guinea pig anti-Synaptohysin-1 (polyclonal)	Synaptic Systems	(Synaptic Systems Cat# 101 004, RRID:AB_1210382)
mouse anti-Bassoon monoclonal (clone SAP7F407)	Abcam	(Abcam Cat# ab82958, RRID:AB_1860018)
Guinea pig anti-vesicular glutamate transporter 1 (VGlut1, polyclonal)	Millipore	(Millipore Cat# AB5905, RRID:AB_2301751)
Guinea pig anti-VAChT (polyclonal)	Synaptic Systems	(Synaptic Systems Cat# 139 105, RRID:AB_10893979)
Alpha-Bungarotoxin - 543	Biotium	00026
Goat anti-Rabbit - 568	ThermoFisher	(Thermo Fisher Scientific Cat# A-11011, RRID:AB_143157)
Goat anti -Rabbit - 488	Jackson Immuno	(Jackson ImmunoResearch Labs Cat# 111-545-003, RRID:AB_2338046)
Goat anti-Mouse - 647	Fisher Scientific	(Thermo Fisher Scientific Cat# A-21235, RRID:AB_2535804)
Goat anti-mouse - IgG1 - 568	Fisher Scientific	(Thermo Fisher Scientific Cat# A-21124, RRID:AB_2535766)
Goat anti-mouse - IgG2a - 568	Fisher Scientific	(Thermo Fisher Scientific Cat# A-21134, RRID:AB_2535773)
Goat anti - Rat - 647	Fisher Scientific	(Thermo Fisher Scientific Cat# A-21247, RRID:AB_141778)
Goat anti-Mouse - 488	Fisher Scientific	(Thermo Fisher Scientific Cat# A32723, RRID:AB_2633275)
Goat anti- Mouse IgG1 - 488	Fisher Scientific	(Thermo Fisher Scientific Cat# A-21121, RRID:AB_2535764)
Goat anti- Mouse IgG2a - 488	Fisher Scientific	(Thermo Fisher Scientific Cat# A-21131, RRID:AB_2535771)
Goat anti- Mouse IgG2a - 647	Fisher Scientific	(Thermo Fisher Scientific Cat# A-21241, RRID:AB_2535810)
Goat anti- Mouse IgG1 - 647	Fisher Scientific	A21246
Goat anti Rabbit - 405	Abcam	(Abcam Cat# ab175652, RRID:AB_2687498)
Donkey anti Rabbit - 405	Abcam	(Abcam Cat# ab175649, RRID:AB_2715515)
Donkey anti Mouse - 543	Biotium	(Biotium Cat# 20305, RRID:AB_2923245)
Donkey anti Rabbit - 543	Biotium	20308
Donkey anti Mouse - 488	Biotium	(Biotium Cat# 20014, RRID:AB_10561327)
Alexa Fluor^®^ 488 AffiniPure Donkey Anti-Guinea Pig IgG (H + L)	Jackson Immuno	(Jackson ImmunoResearch Labs Cat# 706-545-148, RRID:AB_2340472)
Donkey Anti-Rabbit IgG −488	Biotium	(Biotium Cat# 20015-1, RRID:AB_10854232)
Chemicals, peptides, and recombinant proteins		
SMART-Seq v4 Ultra Low Input RNA Kit	Takara Bio	634889
TSMARTer^®^ DNA Unique Dual Index Kit for library Prep	Takara Bio	R400665–R400668
RNasin Plus RNase Inhibitor	Promega	N2615
Cycloheximide	Sigma	C7698-5G
Protease Inhibitor Cocktail	Sigma	P8340-5mL
Heparin sodium salt from porcine intestinal mucosa	Sigma	H3393-50KU
Superscript III First-Strand Synthesis SuperMix	Invitrogen	11752–250
FastStart Universal SYBR Green Master Mix (ROX)	Fisher Scientific	4913850001
M.O.M blocking reagent	Vector Laboratories	27090
Molecular Biology Grade Ethanol	Fisher Scientific	BP2818100
Paraformaldehyde	EMS	15710
Normal Goat serum	Fisher Scientific	16210064
Normal Donkey Serum	Fisher Scientific	NC9624464
Beta Mercaptoethanol	Sigma	M6250
RNeasy RLT buffer	Qiagen	79216
Tissue Plus OCT compound Clear	Fisher Scientific	4585
Deposited data		
Raw RNA sequencing Files	GEO: GSE213987	https://www.ncbi.nlm.nih.gov/geo
Experimental models: Organisms/strains		
*Dlk* ^fx/fx^	(Miller et al. 2009)^28^	
RiboTag: B6N.129-Rpl122tm1.1Psam/J	Jackson laboratories	011029
*ChAT-IRES-Cre*: B6; 129S6-*ChAT*tm(Cre)/Lowl/J	Jackson laboratories	006410
Rosa-tdTomato line (Ai14)	Jackson laboratories	007914
Tmem119-2A-EGFP reporter mouse line	Jackson laboratories	031823
C3 Knockout: B6.129S4-C3tm1Crr/J	Jackson laboratories	029661
Oligonucleotides		
See Table S1		
Software and algorithms		
R	R Foundation for Statistical Computing, Vienna, Austria	R Foundation for Statistical Computing, Vienna, Austria
R.Studio	posit	https://posit.co/download/rstudio-desktop/
GraphPad Prism 9	GraphPad Software, La Jolla, CA	https://www.graphpad.com/scientific-software/prism/
FastQC	Babraham Institute Bioinformatics	https://github.com/s-andrews/FastQC
BBDuK from BBtools	BBMap – Bushnell B.	sourceforge.net/projects/bbmap/
STAR/2.5.2a aligner	(Dobin et al. 2013)^72^	https://github.com/alexdobin/STAR
Samtools	^73^	https://samtools.sourceforge.net/
QoRTs	(Hartley and Mullikin 2015)^74^	https://github.com/hartleys/QoRTs
DESeq2	(Love, Anders, and Huber 2014)^75^	https://github.com/mikelove/DESeq2
ggplot2	(Wickham 2011)^76^	https://github.com/tidyverse/ggplot2
EnhancedVolcano	(Blighe, Rana, and Lewis 2019)^77^	https://github.com/kevinblighe/EnhancedVolcano
DAVID (The Database for Annotation, Visualization and Integrated Discovery)	(“DAVID Functional Annotation Bioinformatics Microarray Analysis”)	https://david.ncifcrf.gov/
GeneSCF	(Subhash and Kanduri 2016)^78^	https://github.com/genescf/GeneSCF
GoPlot	(Walter, Sánchez-Cabo, and Ricote 2015)^79^	https://wencke.github.io/
Volocity Image analysis software (6.3.1)	Perkin Elmer/Quarum Technologies	Volocity 3D Image Analysis Software (RRID:SCR_002668)
Other		
Mm- *Chat* - *C2* (*in situ* probe)	ACD	408731-C2
Mm- *Atf3* - *C3* (*in situ* probe)	ACD	426891-C3
Mm- *C1qb* - C1 (*in situ* probe)	ACD	438101
TSA^®^ Plus Fluorescein	Akoya Biosciences	NEL741001KT
TSA^®^ Plus Cyanine 5 (Cy5)	Akoya Biosciences	NEL745001KT
TSA^®^ Plus Cyanine (Cy3)	Akoya Biosciences	NEL744001KT
RNAscope^®^ Multiplex Fluorescent Reagent Kit V2	ACD	323100
Protein G Magnetic Beads	NEB	S1430S
RNeasy Plus Micro Kit	Qiagen	74034
DAPI Fluoromount-G	Southern biotech	0100–20
Fluoromount-G anti-fade	Southern biotech	0100–35
DynaMag^™^−2 Magnet	ThermoFisher	12321D
ImmEdge hydrophobic pen	Vector Biosciences	H-4000
Csf1r inhibitor PLX5622	Plexxikon	
Chow made with PLX5622 or Control Diet (AIN-76A formula)	Research Diets	
Fine forceps for SNC	Fine Science Tools	11399–80

## Highlights

- DLK signaling in axotomized neurons triggers the loss of upstream synaptic inputs
- DLK regulates the expression of secreted proteins from injured motoneurons
- The activation of complement following nerve injury requires neuronal DLK
- Microglial phagocytosis of synapses is regulated by DLK
