# Supplementary material for: DLK signaling in axotomized neurons triggers complement activation and loss of upstream synapses
Source: Cell Rep. Author manuscript; Available in PMC 2024 May 11. (PMC11088462; doi:10.1016/j.celrep.2024.113801)
Supplement: 1 [file NIHMS1970899-supplement-1.pdf]

Cell Reports, Volume 43

Supplemental information

DLK signaling in axotomized neurons triggers complement activation and loss of upstream synapses

Elham Asghari Adib, Jennifer L. Shadrach, Lauren Reilly-Jankowiak, Manish K. Dwivedi, Abigail E. Rogers, Shameena Shahzad, Ryan Passino, Roman J. Giger, Brian A. Pierchala, and Catherine A. Collins

Supplemental Figure 1

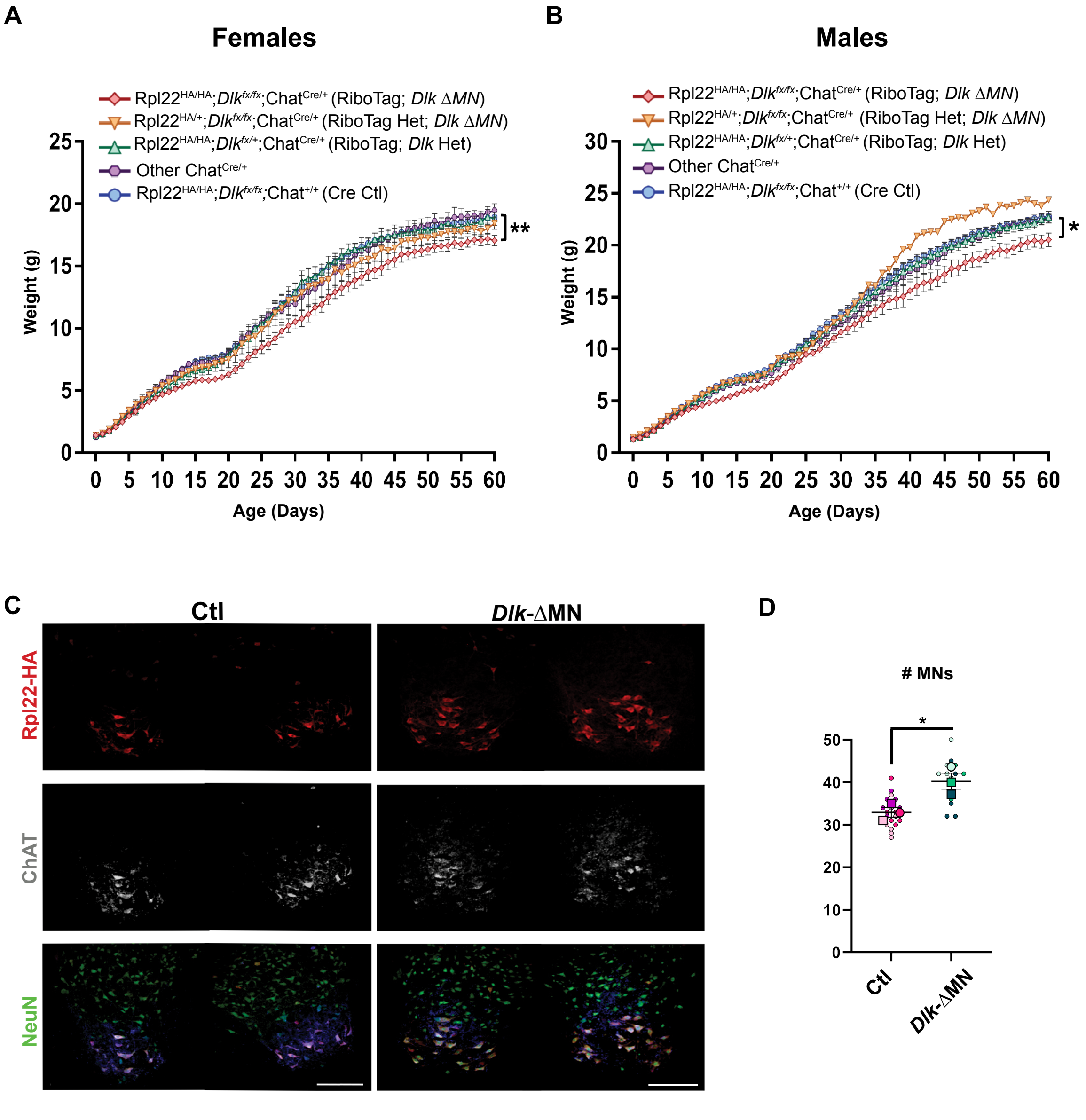

Supplemental Figure 1. *Dlk-ΔMN* animals are healthy despite having slight weight loss.
(A-B) Growth curves of females and males in different genotypes. *Dlk-ΔMN* (RiboTag homozygous; *Dlk fx/fx*; ChAT/+) animals show slightly reduced weight starting at P15. However, these mice are otherwise healthy.
(C) Immunostaining of spinal cords in control and *Dlk-ΔMN* mice for Ribotag (Rpl22-HA), ChAT and NeuN shows that the RiboTag is expressed in motoneurons.
(D) Quantification of motoneuron number in control versus *Dlk-ΔMN* mice.

Supplemental Figure 2

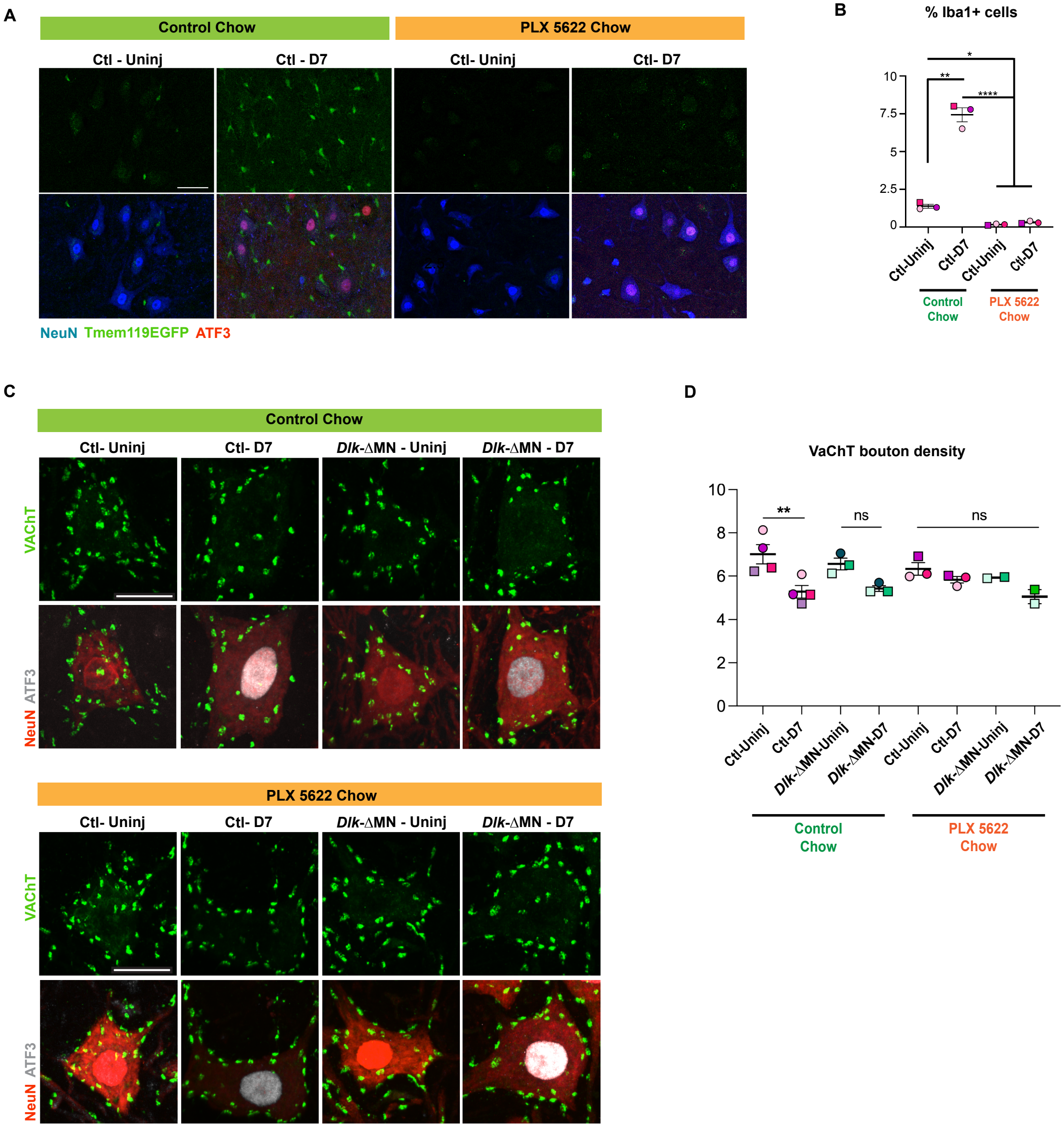

Supplemental Figure 2. Microglial depletion inhibits PNI changes to VAChT bouton density.

A) PLX5622, a Csf1r inhibitor, was used to deplete microglia. Tmem119-EGFP reporter mice were used to endogenously tag microglia. Animals were fed PLX5622 chow, starting 14 days before SNC until tissues were collected D7 after injury. Microglial activation post injury is depleted after feeding on PLX5622. Scale bar is 50 µm.

(B) Quantification of microglia depletion post PLX5622 feeding.

(C) Immunostaining of spinal cords at D7 post SNC on control or PLX5622 chow. Example MN cells bodies are shown with NeuN (red) contacted by VAChT+ boutons (green). ATF3 (gray) was used to identify injured MNs for analysis. Scale bar is 20 µm. Genotypes are *Tmem119*$^{EGFP/EGFP}$ for controls either with PLX5622 or regular chow. *Tmem119*$^{EGFP/EGFP}$;*Dlk*$^{fx/fx}$; ChAT-Cre mice were used for *Dlk-ΔMN* either with PLX5622 or regular chow.

(D) Quantification of mean VAChT bouton density on MN cell bodies. The number of VAChT boutons was measured for all MNs in L3-L6 within the CL (uninjured) and IL (injured) sides of 3 sagittal sections per animal. The mean is shown for each animal. For each MN total count was normalized to the perimeter and reported as per 100µm to estimate bouton density. A One-Way ANOVA with the Tukey test for multiple comparisons was performed. * is p-Value < 0.05 and **** is p-Value < 0.0001.

Supplemental Figure 3

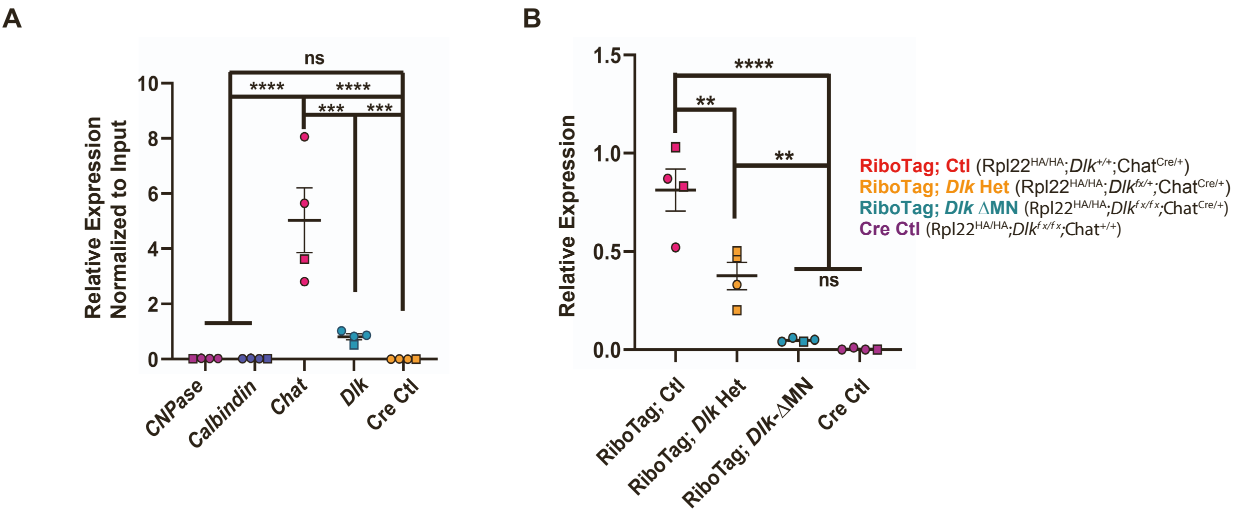

Supplemental Figure 3. RiboTag purification enriches transcripts from MNs.
(A) Relative expression of different transcripts normalized to our input RNA. RiboTag enriches for MN transcripts *Chat* and neuronal transcript *Dlk*, it doesn't enrich transcripts from other cell types such as *CNPase* (Oligodendrocytes) and *Calbindin* (Interneurons).
(B) Relative expression of *Dlk* transcript in WT (*Dlk* +/+), Het (*Dlk fx/*+), and *Dlk fx/flx* (with no Cre) control shows reduction of the transcripts in both Het and *Dlk* KO (*Dlk*-Δ*MN*). One-way ANOVA with the Tukey test was performed. p-value for ** < 0.005, *** < 0.0005, **** < 0.0001.

Supplemental Figure 4

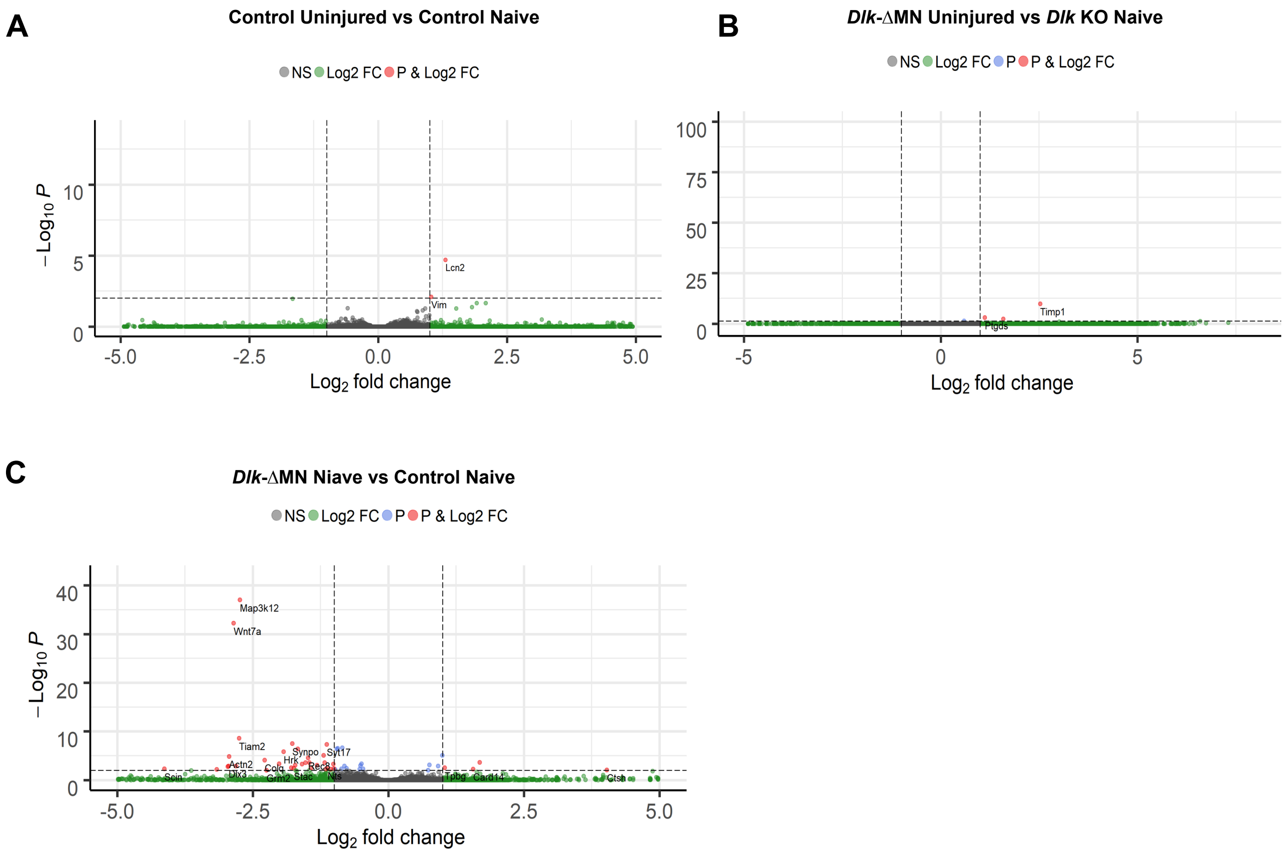

Supplemental Figure 4. Multiple Volcano plots to visualize the differences between multiple conditions.
(A) Volcano plot comparing differentially expressed genes between control, uninjured, and naive conditions.
(B) Volcano plot comparing differentially expressed genes between *Dlk* KO uninjured and naive conditions. There are few genes that are affected in the uninjured side of the spinal cord compared to intact naive mice.
(C) There are not many genes that are different at baseline level between control and *Dlk*-ΔMN condition.

Supplemental Figure 5

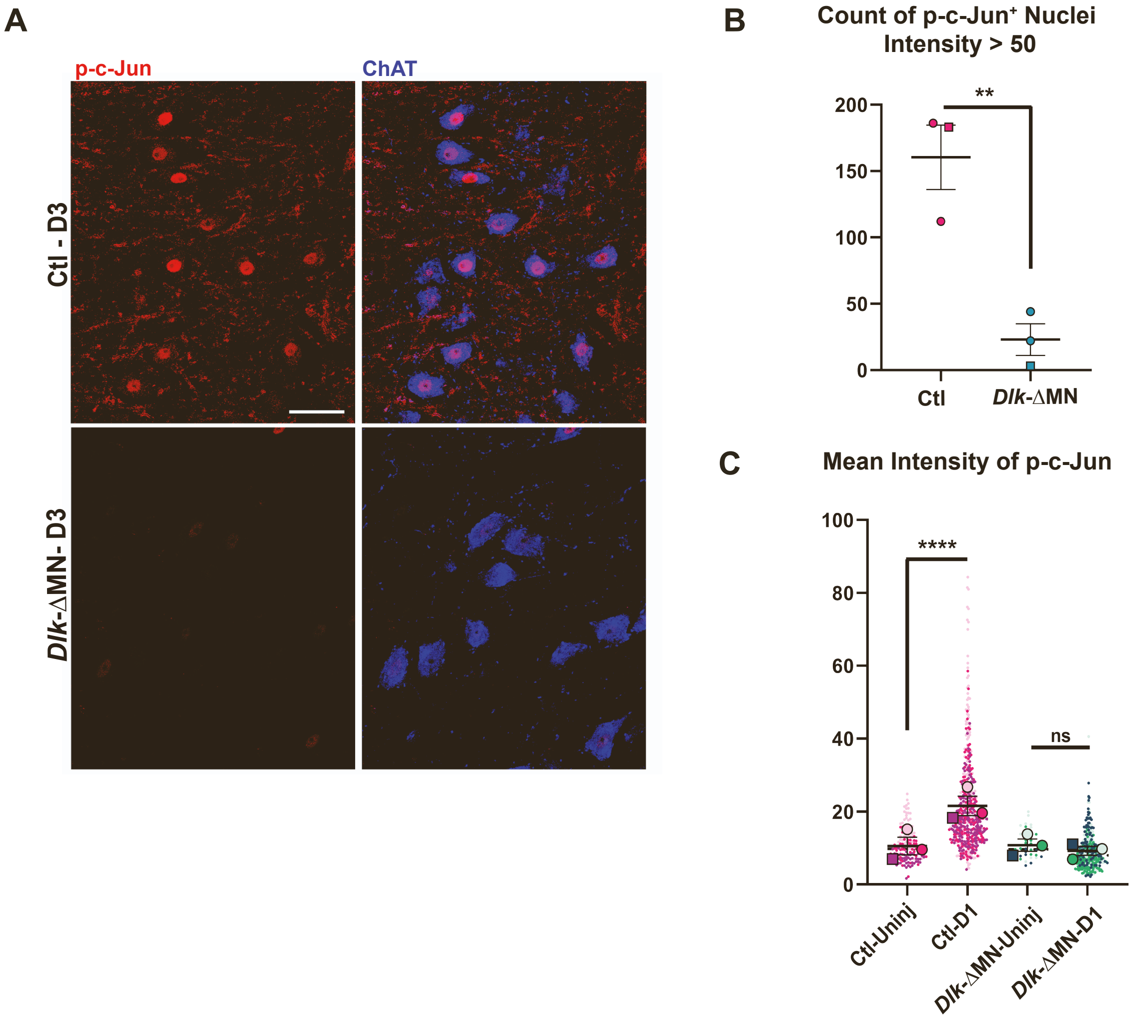

Supplemental Figure 5. Phosphorylation of c-Jun is diminished in *Dlk-ΔMN* mice.
(A) Immunohistochemistry of ChAT (blue, MNs) and p-cJun (red, marker of DLK signaling activation) 3 days post SNC. Control neurons are highly elevated for their p-cjun expression while the *Dlk* KO counterparts are not.
(B) Quantification from A, number of p-cJun positive cells with high intensity is dramatically reduced in *Dlk-ΔMN* animals, 3 days post SNC.
(C) Quantification of mean intensity of p-cJun one day after SNC. There is a 2-fold increase in the mean intensity of p-cJun in control compared to the uninjured, and this increase is not present in *Dlk-ΔMN* animals.
For (B), an Unpaired two-tailed t-test is performed. p-value for ** is 0.0069. For (C) One way ANOVA with Tukey test was performed. P-value for **** is < 0.0001. Genotypes are Rpl22HA/HA; *Dlk-fx/fx* ; (Cre Negative) for controls or Rpl22HA/HA; *Dlk-fx/fx* ; ChAT-Cre for *Dlk-ΔMN.*

Supplemental Figure 6

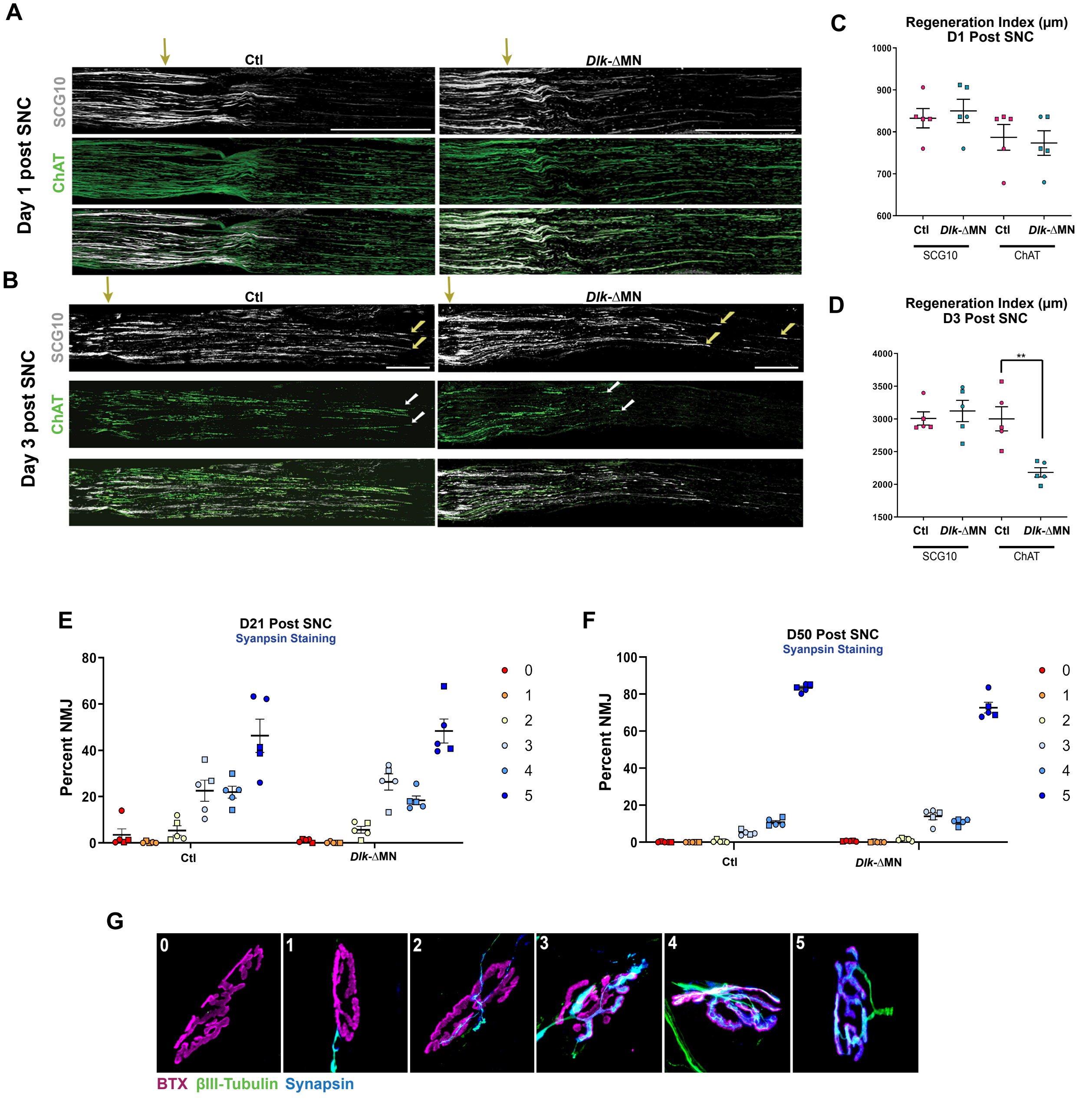

Supplemental Figure 6. Functional regeneration still occurs in *Dlk-ΔMN* animals despite the initial delay.

(A) and **(C)** Immunostaining of sciatic nerves (15 µm section thickness) for SCG10 (gray, new axonal growth), ChAT (green, motor axons) at day 1 **(A)** and day 3 **(C)** post SNC. On day 1, no difference is detected between control and *Dlk* KO regeneration levels but on day 3, regeneration is clearly delayed in *Dlk-ΔMN* animals.

(B) and **(D)** Quantification of regeneration at day 1 and 3 post SNC. Site of injury was defined based on the highest intensity of SCG10 and our knowledge of the proximal and distal end of the axons. 50 µm bins were drawn from the site of injury. Mean intensity of SCG10 was calculated and when the intensity drops to half. The total number of 50µm was used to assess the length of regrowth/regeneration. We consider this number in µm as a regeneration index. For ChAT, we calculate the length from the site of injury until the last ChAT+ tip of axons.

(E) and **(F)** Quantification of percent NMJs that are fully innervated (5) or not innervated at all (0) at day 21 **(E)** and 50 **(F)** post SNC. As seen in the figure, *Dlk-ΔMN* animals can fully regenerate their NMJs after SNC, although initially delayed. One Way ANOVA is performed and p-value for ** is < 0.005. Genotypes are *Rpl22*$^{HA/HA}$; *Dlk*$^{fx/fx}$; Cre Negative for controls or *Rpl22*$^{HA/HA}$; *Dlk*$^{fx/fx}$; *ChAT-Cre* for *Dlk-ΔMN.*

Supplemental Figure 7

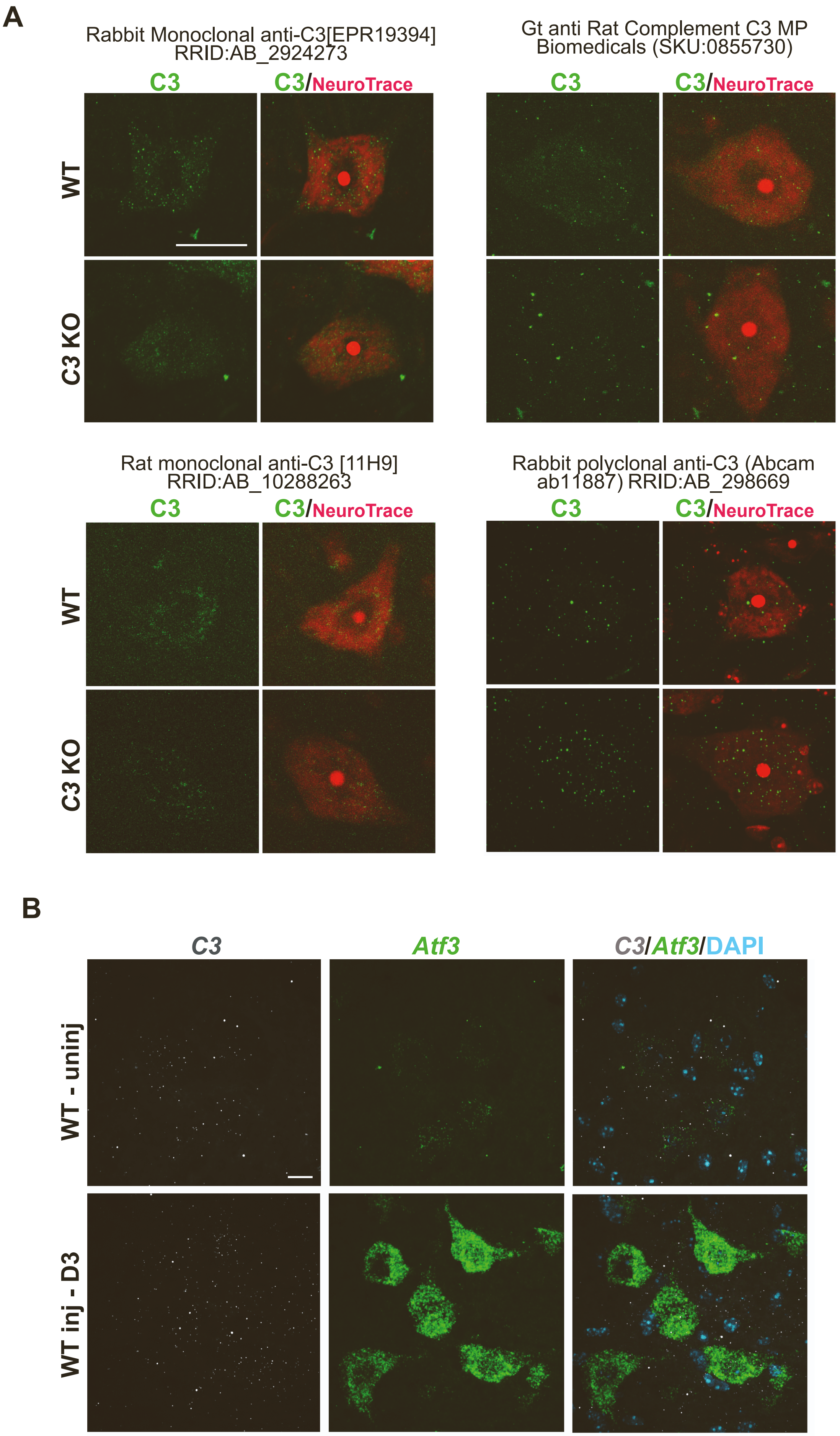

Supplemental Figure 7. Synaptic C3 was not observed surrounding axotomized motoneurons.

(A) Equivalent ipsilateral sections from control and C3 KO mice 7 days post unilateral nerve crush were tested in parallel with antibodies reported to recognize C3b localized to synapses: RRID:AB_2924273 (Rabbit Monoclonal anti-C3[EPR19394], used in [S1]; and Gt anti Rat Complement C3 from MP Biomedicals (SKU:0855730), used in [S2–4]. We also tested RRID:AB_10288263 Rat monoclonal anti-C3 [11H9], and RRID:AB_298669 Rabbit polyclonal anti-C3 (Abcam Cat# ab11887). Not shown, staining on the uninjured contralateral side looked similar for all antibodies. Red channel shows the merge image with NeuroTrace (NT).

(B) RNAscope *in situ* hybridization in the ventral spinal cord 3 days post unilateral nerve crush shows similarly low levels of C3 expression. *Atf3* (green) marks axotomized motoneurons. Scale bars = 20 µm.

Supplemental Figure 8

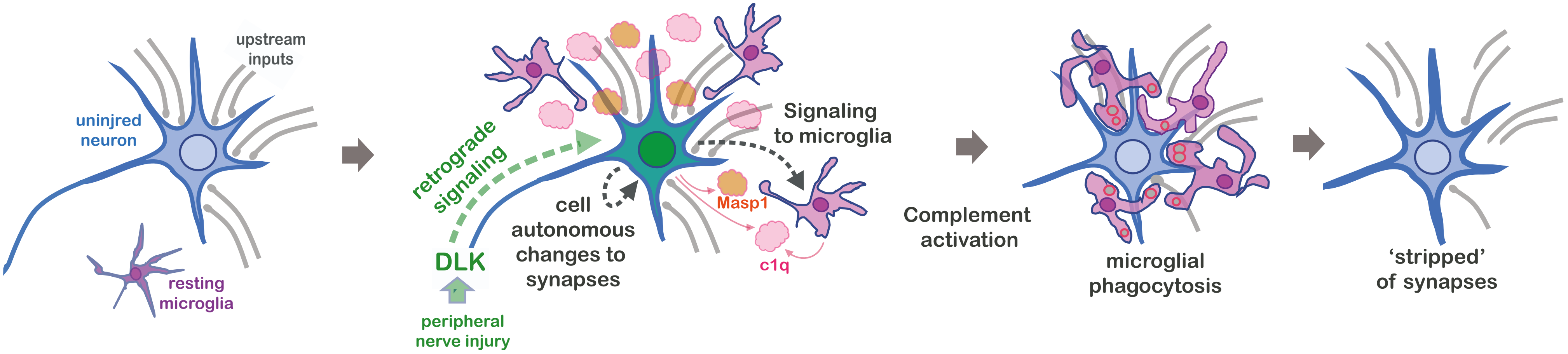

Supplemental Figure 8. Current model for regula-tion of complement activation and synapse loss downstream of DLK activation in motoneurons following peripheral nerve injury.

Supplemental Figure 9

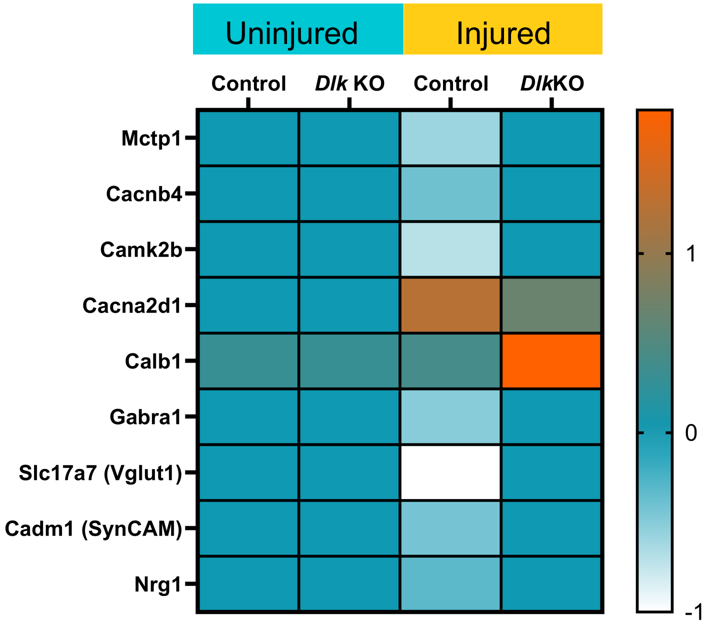

Supplemental Figure 9: DLK-dependent changes in synaptic protein expression in injured MNs.
Heatmaps of selected synaptic genes from the ribosome-associated transcripts in MNs 3 days post SNC. Genotypes are Rpl22HA/HA; *Dlk*+/+ ; ChAT-Cre for controls or Rpl22HA/HA; *Dlkfx/fx* ; ChAT-Cre for *Dlk*-Δ*MN*.

Supplementary Table S1: Primers used in this study

qPCR Primers			
Gene	Primer	Primer Sequence	Source
Calbindin	Forward	GGCTTCATTTCGACGCTGAC	Harvard PrimerBank: 145864481c1
	Reverse	ACGTGAGCCAACTCTACAATTC	
Chat	Forward	CCATTGTGAAGCGGTTTGGG	Harvard PrimerBank: 141802071c3
	Reverse	GCCAGGCGGTTGTTTAGATACA	
CNPase	Forward	ACGAGTGCAAGACGCTATTC	Sanz et al., 2009
	Reverse	CCGCTCGTGGTTGGTATC	
Dlk	Forward	ATTCCTCAGCCATCATCTGG	Made using Primer3
	Reverse	ATTTCGTGGTTTGCTGTTCC	
Rpl22	Forward	GGAGTCGTGACCATCGAACG	Harvard PrimerBank: 31543603c2
	Reverse	CCAGTCTCGGAGGTTGTTCT	
PCR Primers			
Chat	Forward - Ctl	GCAAAGAGACCTCATCTGTGGA	
	Forward - Mutant	CAAAAGCGCTCTGAAGTTCCT	
	Reverse	CAGGGTTAGTAGGGGCTGAC	
RiboTag	Forward	GGGAGGCTTGCTGGATATG	
	Reverse	TTTCCAGACACAGGCTAAGTACAC	
Tdtomato	Reverse	CTCTGCTGCCTCCTGGCTTCT	
	Reverse - Ctl	CGAGGCGGATCACAAGCAATA	
	Reverse - Mutant	TCAATGGGCGGGGGTCGTT	
Dlk	Forward	GGAAAGGTGTGGCCCTGGCTGGCTTGGAAG	
	Reverse	CAGGTGCAGCAAGATCTGTCGGAATGATGG	
Tmem-EGFP	Forward-Ctl	GTCAGGAGGAGGCCCAGGAA	
	Forward-EGFP	CTGCTGCCCGACAACCACTA	
	Reverse	GTTTCCTGGGGTGCACCAGA	

References in Supplemental Information:

S1. Zhong, L., Sheng, X., Wang, W., Li, Y., Zhuo, R., Wang, K., Zhang, L., Hu, D.-D., Hong, Y., Chen, L., et al. (2023). TREM2 receptor protects against complement-mediated synaptic loss by binding to complement C1q during neurodegeneration. Immunity *56*, 1794–1808.e8. 10.1016/j.immuni.2023.06.016.

S2. Schafer, D.P., Lehrman, E.K., Kautzman, A.G., Koyama, R., Mardinly, A.R., Yamasaki, R., Ransohoff, R.M., Greenberg, M.E., Barres, B.A., and Stevens, B. (2012). Microglia sculpt postnatal neural circuits in an activity and complement-dependent manner. Neuron *74*, 691–705. 10.1016/j.neuron.2012.03.026.

S3. Werneburg, S., Jung, J., Kunjamma, R.B., Ha, S.-K., Luciano, N.J., Willis, C.M., Gao, G., Biscola, N.P., Havton, L.A., Crocker, S.J., et al. (2020). Targeted Complement Inhibition at Synapses Prevents Microglial Synaptic Engulfment and Synapse Loss in Demyelinating Disease. Immunity *52*, 167–182.e7. 10.1016/j.immuni.2019.12.004.

S4. Shi, Q., Colodner, K.J., Matousek, S.B., Merry, K., Hong, S., Kenison, J.E., Frost, J.L., Le, K.X., Li, S., Dodart, J.-C., et al. (2015). Complement C3-Deficient Mice Fail to Display Age-Related Hippocampal Decline. J. Neurosci. *35*, 13029–13042. 10.1523/JNEUROSCI.1698-15.2015.
